# Mining anion–aromatic interactions in the Protein Data Bank†

**DOI:** 10.1039/d2sc00763k

**Published:** 2022-03-01

**Authors:** Emilia Kuzniak-Glanowska, Michał Glanowski, Rafał Kurczab, Andrzej J. Bojarski, Robert Podgajny

**Affiliations:** Faculty of Chemistry, Jagiellonian University Gronostajowa 2 30-387 Kraków Poland robert.podgajny@uj.edu.pl; Jerzy Haber Institute of Catalysis and Surface Chemistry, Polish Academy of Sciences Niezapominajek 8 30-239 Kraków Poland; Maj Institute of Pharmacology, Polish Academy of Sciences Smętna 12 31-343 Kraków Poland bojarski@if-pan.krakow.pl

## Abstract

Mutual positioning and non-covalent interactions in anion–aromatic motifs are crucial for functional performance of biological systems. In this context, regular, comprehensive Protein Data Bank (PDB) screening that involves various scientific points of view and individual critical analysis is of utmost importance. Analysis of anions in spheres with radii of 5 Å around all 5- and 6-membered aromatic rings allowed us to distinguish 555 259 unique anion–aromatic motifs, including 92 660 structures out of the 171 588 structural files in the PDB. The use of a scarcely exploited (*x*, *h*) coordinate system led to (i) identification of three separate areas of motif accumulation: A – over the ring, B – over the ring-substituent bonds, and C – roughly in the plane of the aromatic ring, and (ii) unprecedented simultaneous comparative description of various anion–aromatic motifs located in these areas. Of the various residues considered, *i.e.* aminoacids, nucleotides, and ligands, the latter two exhibited a considerable tendency to locate in region A*via* archetypal anion–π contacts. The applied model not only enabled statistical quantitative analysis of space around the ring, but also enabled discussion of local intermolecular arrangements, as well as detailed sequence and secondary structure analysis, *e.g.* anion–π interactions in the GNRA tetraloop in RNA and protein helical structures. As a purely practical issue of this work, the new code source for the PDB research was produced, tested and made freely available at https://github.com/chemiczny/PDB_supramolecular_search.

## Abstract 2

The comprehensive analysis of non-redundant PDB macromolecular structures investigating anion distributions around all aromatic molecules in available biosystems is presented.

## Graphical abstract

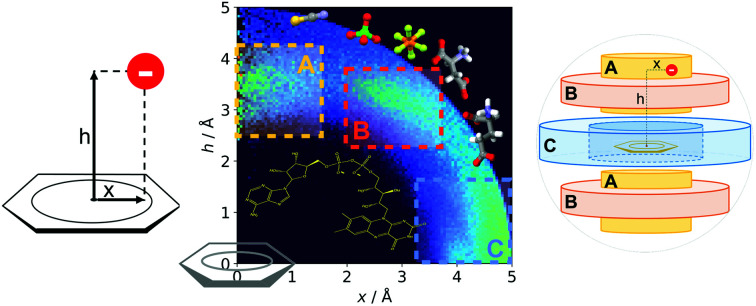

## Introduction

Non-covalent interactions that involve anions and aromatic rings have become a focal point in the field of supramolecular chemistry, as they continue to stimulate the exploration of functional molecular materials and studies of molecular activity in biological systems. The various underlying interaction modes (synthons) rely on a multitude of electron density distribution schemes, which depend on side substituents (electron withdrawing and donating groups) and heteroatoms (N, S, *etc.*) in the aromatic ring. The resulting quadrupole moment decides whether an anion tends to locate in the space above the ring (positive, π-acidic surface) or instead in more distant peripheral regions closer to the ring plane (negative, electron-poor edge). Thus, canonical anion–π synthons reveal their significance in performance of advanced small-molecule catalytic systems dedicated to specific organic reactions,^1–3^ photophysical systems based on charge or electron transfer properties,^4–9^ anion recognition, binding, and sensing,^10–13^ anion transport,^14–17^ or anion directed self-assembly of polynuclear coordination complexes.^18^ Further on, the new generation supramolecular and coordination anion–π architectures hosting mononuclear^8,19–21^ and polynuclear d-metalate complexes^9,22,23^ were recently reported in the context of anion binding,^19,20^ molecular crystalline composites,^21^ charge transfer and photophysical properties,^8,9,20–22^ or magnetic properties.^23^ In parallel, edgewise cooperative synthons that exploit multiple side _ring_C–H⋯anion contacts at the ring edge are well known to stabilise numerous molecular architectures.^24^ The above distinction is also relevant to biological systems, and the significance of the representative modes has been a topic of debate over the recent decade in the context of enzymatic activity improvement,^25,26^ ligand or active site stabilization,^27–32^ secondary structures and folding,^33–36^ and proteins behaviour in membrane and extramembrane environments.^37^ For example, the edgewise positioning of aspartate and glutamate anionic groups near phenylalanine, tyrosine, or tryptophan in proteins occurs due to recently recognised _ring_C–H⋯anion interactions,^26,37–40^ which act as an alternative to canonical salt bridges. In contrast, some enzymatic processes have been shown to be controlled by the locations of anions and other entities in the space over an aromatic ring with clear positive electrostatic potential. Examples include the hydroxylation of uric acid to (*S*)-allantoin by urea oxidase,^27,31^ inhibition of malate synthase activity by phenyl-diketo acids,^30^ and flavine-dependent co-enzyme activity during sulphide oxidation and electron transfer.^29,41,42^ Several review articles have systematised correlations between the geometries, energies, and biological roles of the underlying motifs collected in the Protein Data Bank (PDB). These articles have focused mainly on F^−^, Cl^−^, Br^−^, I^−^, SO_4_^2−^, PO_4_^3−^, NO_3_^−^, CO_3_^2−^, Asp, and Glu localised in the neighbourhoods of amino acids and nucleobase aromatic rings.^38,43–45^ In the broad context of the above debate, this paper introduces the first comprehensive analysis of non-redundant PDB macromolecular structures investigating anion distributions around all aromatic molecules in available biosystems (including ligands, *i.e.* molecules other than amino acids or nucleotides). We used a generalised set of anions and an alternate methodology to indicate the importance of the chosen coordinate system in such analyses and its influence on perception of the results. We hope that our studies enable the modernisation and generalisation of available knowledge regarding anion spatial distributions and anion interactions with aromatic rings. This should provide an improved means of approaching the analysis and representation of their occurrence frequency and thus enable their discussion in the context of various systems and processes.

## General methodology and data treatment

**Fig. 1:**
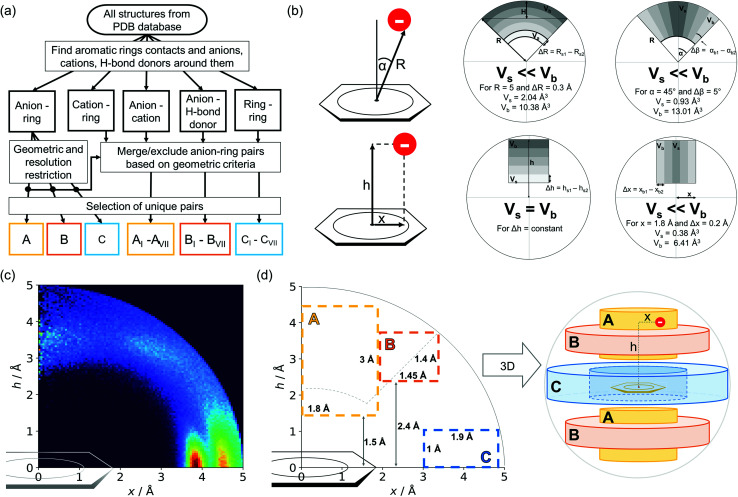
(a) Simplified methodological scheme. The blocks at the bottom of the scheme represent various groups of synthons described in the text. (b) Two different coordinate systems might be used to describe chemical individual around the aromatic species of interest ((*R*, *α*) (top) and (*x*, *h*) (bottom)). For a detailed explanation, see the ESI.† (c) A two-dimensional histogram calculated for the non-redundant set of macromolecules (resolution better than 2.5 Å, 50 086 sequence clusters) shows the anion density in the neighborhood of the tested aromatic ring. Each pixel shown in the chart represents the number of anions in the histogram bin divided by its volume (the bin is a small cylindrical shell designated by *x* and *h*, bin size: Δ*x* = Δ*h* = 0.05 Å). Histograms of distribution and density for all structural models (independent of resolution and method) are available as Fig. S8 in the ESI.† To visualise the bin concept, compare this to the (d) schematic representation of defined regions A, B, and C (see also Fig. S7†). The grey dotted line represents fragments of space explored by Bauzá and coworkers.^43^ The volumes of regions A, B, and C are 13.1%, 13.8%, and 20.2%, respectively, of the 5 Å radius sphere (approximately 70% of anions locate in regions A–C).

Almost all of the 171 588 structural cif files from the Protein Data Bank^46–48^ (access 29.11.2020) were analysed using our Python program.^49^ A PDB submodule^50^ from the Biopython package^51^ was used to read and parse the files. The procedure for finding unique anion–ring pairs and assisted interactions was as follows (see Fig. 1a). In the initial step, all aromatic rings were detected (see Aromatic ring detection in the ESI†). Then, for each ring, a sphere with a 5 Å radius around the ring's centroid was explored for the presence of anions (Anion classification in the ESI†), cations (Cation classification in the ESI†), and other aromatic rings and methyl groups from aliphatic amino acids: valine, threonine, alanine, leucine, and isoleucine. If an anion was found, H-donors were searched for (Hydrogen bonds in the ESI†). Structural models with resolutions better than or equal to 2.5 Å were used in further analyses. In 118 805 structural files that met the resolution criteria, we found 555 259 unique cases where an anion was present within a sphere with a 5 Å radius around the aromatic ring (92 660 structures). To identify non-redundant interactions, we assigned macromolecular chains to sequence clusters^52,53^ and processed only unique pairs (Unique records in the ESI†). We used the height over the ring plane (*h*) and the distance between the centroid of the ring and the anion projection onto the ring plane (*x*) to describe anion positions in the sphere around the aromatic ring (Fig. 1b). Unlike the great majority of previous papers, we did not use the typical (*R*, *α*) coordinate system because, in this system, attempts to analyse the distributions of chemical individual with respect to angle or distance from the ring lead to division of the spherical cone into equidistant slices and therefore to comparison of slices with unequal volumes (compare to Fig. 1b and the detailed explanation in Fig. S2–S7 within the ESI†). The natural consequence is that the more distant the point being considered is from the ring (larger analysed volume), the more anions are found.^43^ In our opinion, this inherent “feature” of the (*R*, *α*) coordinate system should be emphasized, as this system is used widely in analysis of chemical entities' statistical distributions (including for anions, cations, and hydrogen bonds). Although it is quite useful, one should carefully reflect on the consequences of the coordinate system used. The findings were processed and analysed using Pandas,^54,55^ the Matplotlib^56^ packages, and the Pymol^57^ program. As a result, we could visualise anion distributions and anion densities around aromatic rings in macromolecules (Fig. 1c). A few characteristic areas are conspicuous in this representation, so we decided to distinguish and carefully analyse three regions (Fig. 1d). The first region was related to anions localised above and below the ring skeleton, which might be engaged in anion–π interactions (marked as yellow rectangle, A). The second (blue rectangle, C) corresponded to anions localised roughly in the plane of the ring. The third region was the space between regions A and C (orange rectangle, B), where anions were located above the ring substituent. The regions above were distinguished independently within our study and corresponded nicely to the most frequent anion locations observed within the overall anion⋯aromatic motifs (see Introduction). It is important to note that approximately 70% of anions in the collection considered were located within these regions.

## Results and discussion

### Dependence of anion distributions on the aromatic ring and anion

**Fig. 2:**
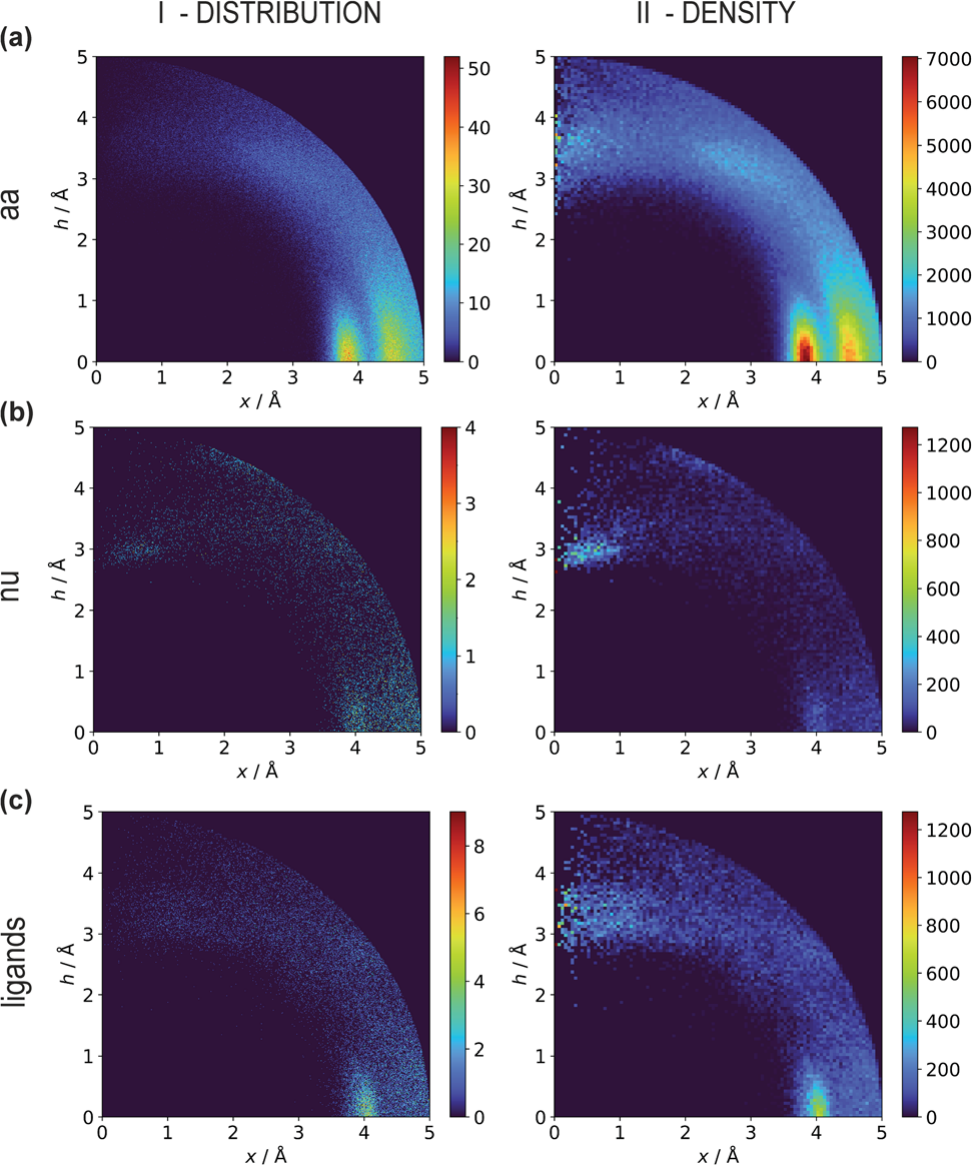
Two-dimensional histograms of anion locations around the rings as functions of *x* and *h*. In column I, the histograms present the number of anions (distribution, bin size: Δ*x* = Δ*h* = 0.01 Å) whereas column II presents local anion densities (density, bin size: Δ*x* = Δ*h* = 0.05 Å; when *x* ≪ 1 Å, the bin volume is close to 0; this explains the small number of red pixels (high density) near the *h* axis). Histograms for aromatic amino acids (aa) (a), nucleotides (nu) (b), and other aromatic ligands (c) are presented in subsequent rows (with no anion restrictions). Compare to Tables S1–S3.†

Two types of charts are presented for visualisation of anion distributions around various aromatic rings (Fig. 2). On the distribution charts in column I, each pixel represents the number of anions in a bin (the concept of a bin is explained in Fig. 1), whereas in column II each pixel represents the anion density, which results from dividing the number of anions in a bin by the bin volume; volume scaling enables comparison of different bins. The ratios of the occurrences of the most common residues (anions and rings) in regions A, B and C to their occurrences in the entire sphere are presented in Tables S4 and S5.† This parameter indicates the positioning preferences of various residues.

#### Amino acid quadrupoles

Aromatic amino acids are dominated by interactions in the ring plane (C), where two strong maxima are visible. This is an effect of the numerous charge-assisted hydrogen bonds between aspartic (Asp) and glutamic (Glu) acids and the hydrogen-bond donors commonly present in proteins. The first maximum near 3.8 Å corresponds to histidine (His–Asp pairs: 39 666, His–Glu: 37 416) and tryptophan (Trp–Asp: 11 946, Trp–Glu: 13 432), whereas the second, which is less intense, corresponds mostly to tyrosine (Tyr–Asp: 35 729, Tyr–Glu: 38 335). However, regions A and B are also clearly distinct on the density plot. Interestingly, His is the most common in all regions, even though its abundance in proteins is lower than for Phe or Tyr^58^ (which was confirmed in our dataset).

**Fig. 3:**
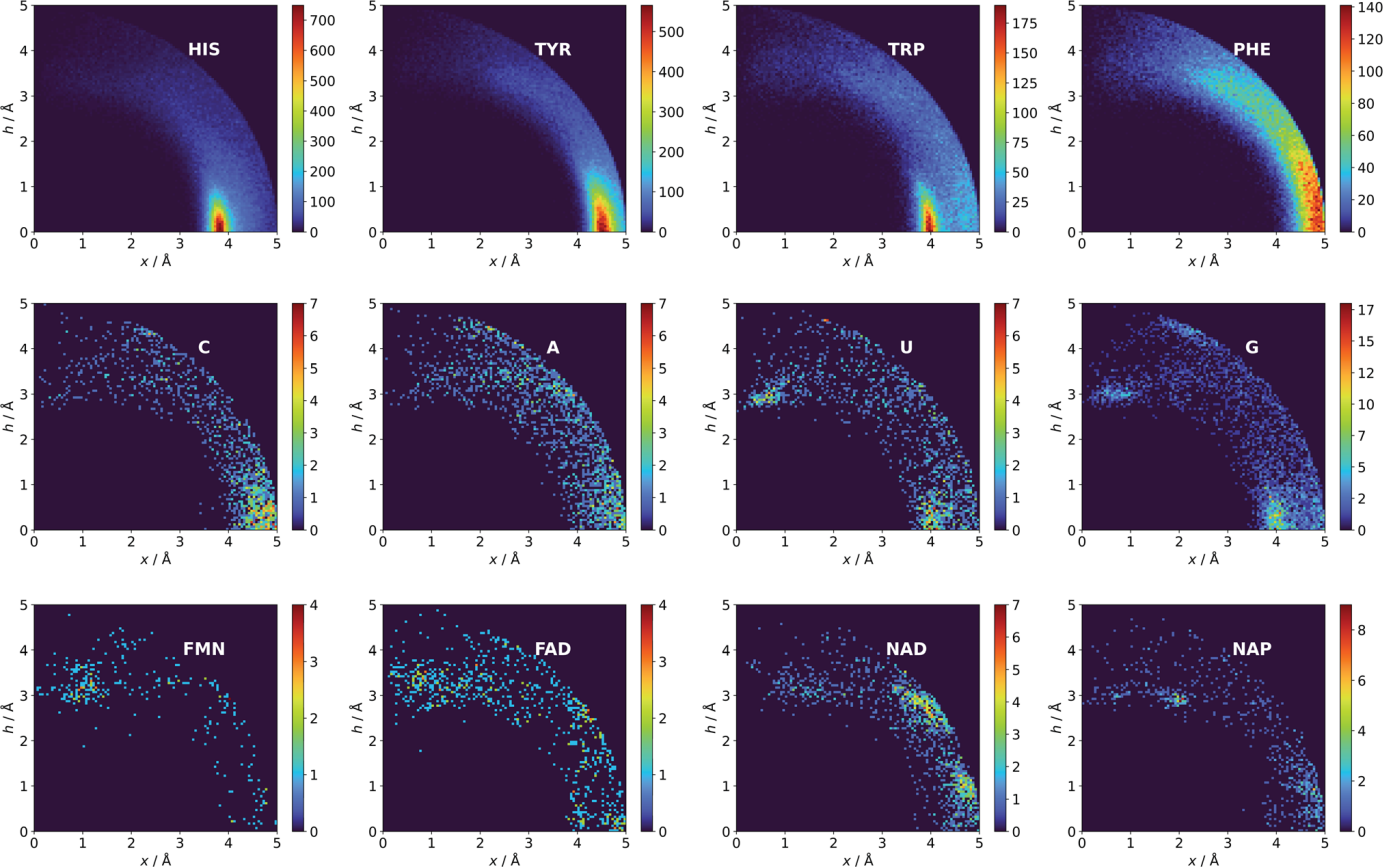
Anions distributions around the most common aromatic rings (bin size: Δ*x* = Δ*h* = 0.05 Å). Compare to the distribution of the most common anion (Fig. S10†). Note that the presented histograms show the anion distribution, not the anion density. C, A, U, and G are the sums of the respective RNA and DNA nucleobases.

There might be several reasons for this. First, His is the smallest aromatic amino acid and therefore is more mobile than other AAs; second, His can be protonated easily under physiological conditions. This might increase its electrostatic contribution and influence its ability to form salt bridges. Although Tyr is less abundant than Phe, it locates more often in the anion neighbourhood (compare to Table 1 and Fig. 3). This is because it can form hydrogen bonds and has a larger quadrupole moment. The percentages of Glu and Asp anionic groups located in region A are almost the same: 6 and 6.1%, respectively. Glu superiority is observed in the B region (19.4 *vs.* 14.4%), whereas 54.4% of Asp and 48.5% of Glu locate in region C. These differences might correspond to the lengths of the Asp and Glu sidechains. All aromatic AAs strongly prefer anion localisation in regions C or B; fewer than 8% of the anions of each AA are present in region A.

(a) The number of anions localized in A–C regions over the given aromatic ring. (b) The number of the given anions localised in A–C regions. The percentages represent the ratio of the number of pairs in a region to the number in the entire sphere. For more detailed information, see Tables S1–S5 and Fig. S9, S10 in the ESI

**Table:** 

(a) Quadrupoles	A	%	B	%	C	%
PHE	6499	7.1	19 280	21.2	24 628	27.0
TYR	6663	4.3	19 478	12.5	86 722	55.3
HIS	9442	6.4	24 296	16.1	102 736	62.9
TRP	5027	7.8	11 209	17.4	30 402	46.4

**Table:** 

(b) Anions	A	%	B	%	C	%
ASP	10 921	6.1	26 309	14.4	103 914	54.4
GLU	12 437	6.0	40 320	19.4	105 467	48.5
ALL	31 550		79 916		260 147	

#### Nucleotide quadrupole

Nucleotides are treated both as quadrupoles (purine and pyrimidine) and as anions (phosphate groups). In this case (Fig. 2b), such distinct dominance of anions in the ring plane is not observed. In general, ribonucleotides (RNUs) interact preferentially with each other instead of with AAs or other anions, unlike deoxyribonucleotides (DNUs), which interact mostly with amino acids (see Tables S1–S3†). Ribonucleotides are represented more frequently than DNU, but the ratio defined by DNU over all NU increases from region A to region C (from 8 to 20% for aromatic rings, and from 18 to 43% for anions) (see Table 2). Unlike AA and DNU, RNU rings exhibit a greater tendency to localise anions in region A (Table S5†). For instance, 25.4% of the uracil rings interact with anions localised in region A. The anionic groups of adenine and guanine localise above other aromatic rings with comparable frequencies (15.7 and 16.1%, respectively, see Table S4†). Differences between nucleotides might be explained by the shape of the electrostatic potential (ESP) and the polarizability.^59^

**Table:** The number of nucleotides in anion–ring pairs, distinguished by quadrupole and anion. We use standard abbreviations, *e.g.* A – adenine nucleotide, DA – adenine deoxyribonucleotide

	Region A		Region B		Region C	
	Anion	Ring	Anion	Ring	Anion	Ring
**RNU**						
A	472	198	472	385	1419	464
G	306	459	261	408	750	1494
C	122	115	247	199	542	643
U	153	337	226	212	687	559

**DNU**						
DA	57	25	115	72	598	70
DG	81	18	133	92	696	217
DC	51	11	130	69	590	409
DT	42	43	163	61	638	95

#### Ligand interactions

We find 13 883 ligands (5035 anions and 8848 quadrupoles different than AA and NU) that are part of anion–aromatic ring pairs. Although the majority of ligands occur only once, some quadrupoles and anions are common (aromatic: flavin-adenine dinucleotide (FAD), flavin mononucleotide (FMN), nicotinamide-adenine-dinucleotide (NAD), nicotinamide-adenine-dinucleotide phosphate (NAP), adenosine-5′-tri/diphosphate (ATP/ADP), guanosine-5′-diphosphate, vitamin B6 phosphate (PLP), protoporphyrin IX containing Fe (HEM), dihydro-nicotinamide-adenine-dinucleotide phosphate, imidazole, heme C, 1,4-dihydronicotinamide adenine dinucleotide) (anions: SO_4_^2−^ (SO4), acetate (ACT or ACY), Cl^−^ (CL), Br^−^ (BR), I^−^, PO_4_^3−^ (PO4), formate (FMT), NO_3_^−^ (NO3), citrate, malonate (MLI), HEM, NAP, and ATP). Anions around aromatic ligands are distributed in all regions A–C, with a blurred maximum in C at approximately 4 Å (Fig. 2c, 3 and S10†). The preferable height *h* above the ring is in the 2.8–3.8 Å range. We find 2714, 4155, and 11 708 anion–ring pairs in regions A, B, and C, respectively, where the ring is neither AA nor NU. Similarly, we find 6527 (A), 10 809 (B), and 42 296 (C) pairs where the anion is different from AA and NU. Approximately 20–25% of small carboxylic acids located near the ligand rings are present in region A (FMT (23.3%), ACT/ACY (20.7%), MLI (22.6%)). FMN and FAD exhibit strong tendencies to position anions above the ring (in region A). This is indicated by the decreasing region : sphere occurrence ratios (FMN decreases from 57.1 in A to 11.4% in C and FAD decreases from 36.2 to 19.6%).

The above result is an effect of notable positive quadrupole moments and diverse ESP surfaces along with the π-conjugated skeletons of FAD and FMN.^29^ In 2017, Freitas and Schapira presented analyses of the most common ligand interactions, such as hydrophobic interactions and hydrogen bonds. However, anion–π interactions were not considered.^60^ Their studies reveal that cation–π interactions are much less common than others. We find it likely that more ligands engage in anion–π interactions than in the cation–π interactions reported by Freitas and Schapira. Nevertheless, we are aware of differences in methodology.

#### Comparison of AAs, NUs, and ligands

**Fig. 4:**
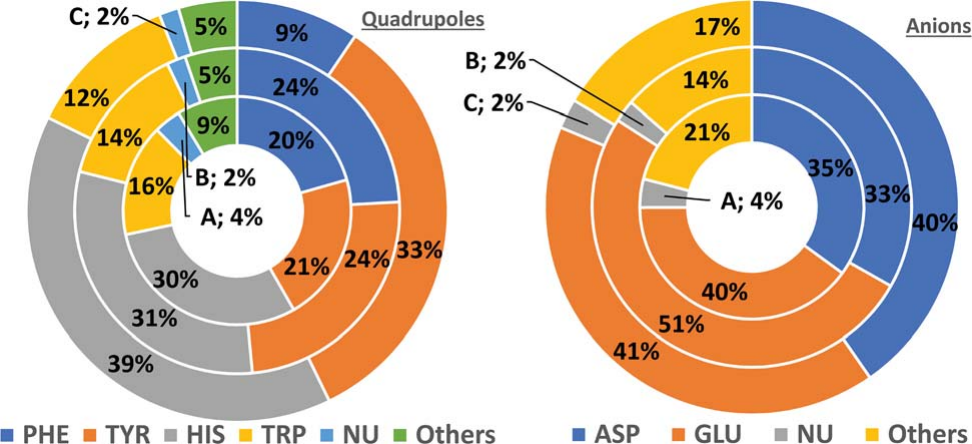
Pie charts present the proportional distributions of quadrupoles and anions in regions A–C.

In general, the number of found pairs increases from region A, through B, to C for all analysed residues. However, the proportions of quadrupoles and anions change significantly (compare to Fig. 4). Phe and Tyr occur in almost identical percentages in areas A and B (20–21 and 24% for regions A and B, respectively). At the same time, Tyr represents one-third of all quadrupoles in area C, whereas Phe makes up just 9%. The fraction of His increases from 30% in region A to 39% in region C. In the case of anions, interesting trends can be observed with Asp and Glu. Although Glu is more abundant in proteins^58^ (which was confirmed in our dataset), their fractions in region C are almost identical (41 and 40%, respectively). Glu dominates (51% *vs.* 33%) in region B and is represented slightly more than Asp (40% *vs.* 35%) in region A. This might be caused by the longer side chain of Glu. Notably, the fractions of quadrupole nucleotides (2, 2, and 4% for regions C, B, and A, respectively) as well as non-standard aromatic molecules (5, 5, and 9% for regions C, B, and A, respectively) increase from C to A.

### Structure type

The majority of found pairs are localised in proteins: 93% for A and 95% in B and C (most of the structures available in PDB are proteins). The trends of the ribonucleotide-containing macromolecule fractions in areas A–C are interesting. The general conclusion is that the fractions of RNA and DNA increase from region C to region A (including any protein–RNA or DNA complexes). To be clear, our algorithm assigns polymerase DNA as a protein–DNA complex, and does the same for any protein with even one nucleotide docked and *vice versa*. Nevertheless, about 80% of pairs (in protein–RNA complexes) found above the ring (A) consist of nucleotides, whereas in the ring plane (C), about half of the pairs in such complexes consist of amino acids. More information regarding structural statistics is available in Table S6.†

### Anion–ring pair in sequence—secondary structure correlations

**Fig. 5:**
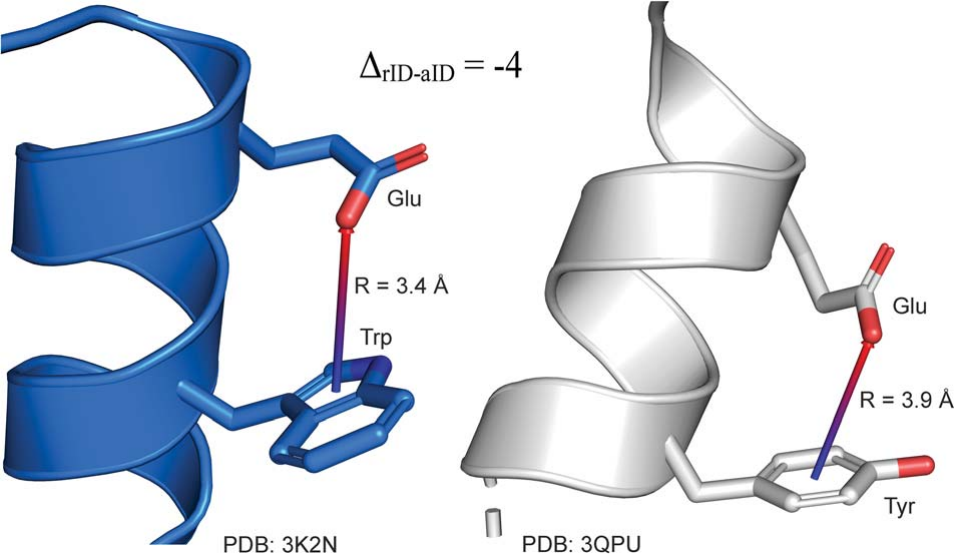
Example of an anion–π interaction between residues *i* − 4.^88–90^

**Fig. 6:**
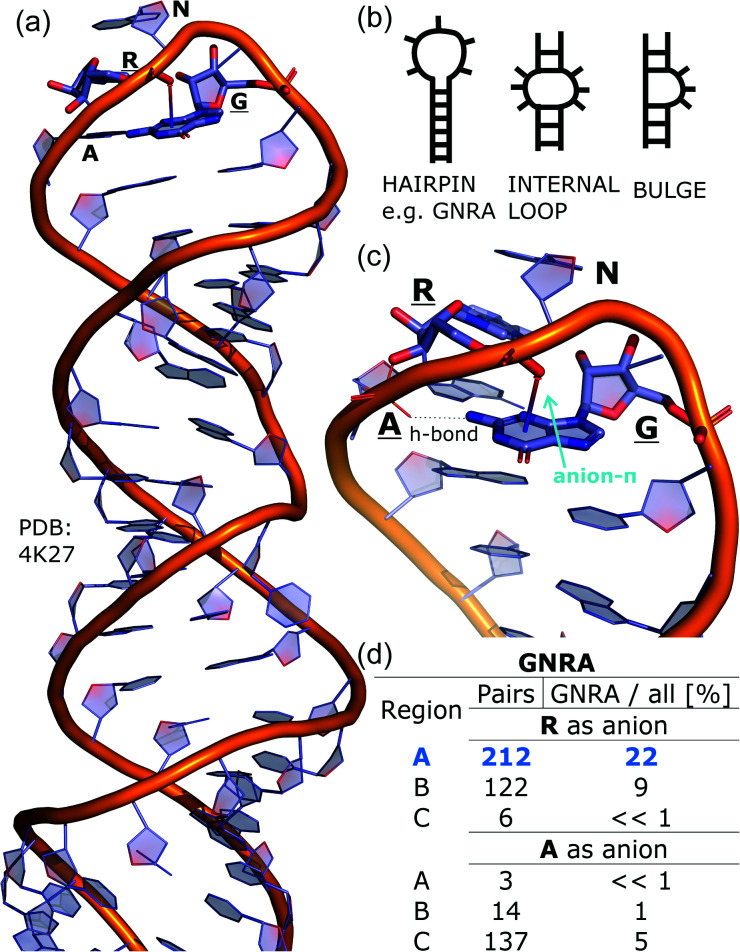
(a) The three-dimensional structure of the duplex form of RNA with a GNRA hairpin at its end (PDB code: 4K27).^91,92^ (b) Schematic representation of typical RNA secondary elements, where the oxygen atom from the anionic phosphate backbone is located in region A or B above the aromatic ring. (c) Close view of the hairpin from 4K27. (d) Anion locations in the GNRA motifs.

To determine whether there are any repeated motifs in sequences correlated with anion location around the ring and secondary structure, we analysed anion–quadrupole pairs where both residues belong to the same chain. The secondary structure type was assigned using the DSSP program.^61,62^ For each region A, B, and C, we prepared histograms of occurrence in the function of difference between aromatic ring and anion identifiers—*Δ*_rID–aID_ (rID – ring number in sequence, aID – anion number in sequence) (Fig. S11†). In proteins, anions located in regions A and B are bonded directly to aromatic amino acids more frequently, whereas a distance of more than one residue between the anion and the ring favours hydrogen-bond formation in region C. Although the most frequent pairs are those where the *Δ*_rID–aID_ are less than 10 residues for each region, the relative frequencies of more distant pairs are significantly higher in region C. An extraordinary number of pairs with *Δ*_rID–aID_ = −4 are observed in regions A and B. The majority of these are related to two subsequent turns in the α-helices (3.6 residues per turn) (Fig. 5 and Table S7†). In many such cases, the anion points into an aromatic ring even if there is enough space for other conformations. This may suggest a role for anion–π interaction in stabilization of α-helices. This conclusion should be supported by appropriate calculations, however, this is not the aim of this work. In their extensive analysis of short contacts between planar AA side chains, Waters, Bhattacharyya, and Chakrabarti noticed that a *Δ*_rID–aID_ = −4 between interacting residues (especially aromatic AAs) is observed commonly in helical structures.^63,64^ They presented extensive valuable information that indicated possible interactions between them. However, this observation was not linked to possible anion–π interactions. Sequence analyses of the most common amino acid pairs are presented in the ESI (Fig. S12 and S13†). The majority of examples indicate vaguely symmetrical distributions, which means there is no significant preference for the quadrupole to be before or after the anion. However, a few representative protein chain motifs exhibit such order preferences. For example, in the Glu–Phe and Glu–Tyr dimers (*Δ*_rID–aID_ = 1) found in regions A and B, the carboxylic group of Glu is located above the Phe or Tyr ring. Such dimers tend to be observed in α-helices, π-helices (often as the first or last turn), and loop bends that include hydrogen-bonded turns (Table S7†). The opposite (*Δ*_rID–aID_ = −1) dimers Phe–Glu and Tyr–Glu are several times rarer. It is worth noting that even if a sequence motif appears to be favourable (Fig. S11†), it is not equal to any structural pattern. For example, although the His–x–x–Glu (where x is any amino acid and *Δ*_rID–aID_ = −3) motif seems to be relatively frequent in region A, we could not assign a specific pattern to the secondary structure. On the other hand, we suspect that the anionic groups have interesting roles in the formation of the GNRA motif (G – guanine, N – any nucleotide, R – guanine or adenine, A – adenine) of the RNA hairpin secondary structure. GNRA is among the most widespread and well-researched RNA tetraloops.^65–68^ In this motif the helical form of RNA (duplex) is unfolded to create a bent phosphate backbone and an unpaired nucleotide loop (see Fig. 6). GNRA is stabilised by the hydrogen-bond network and might be supported by aromatic ring stacking. Nevertheless, we find that in region A, motif *Δ*_rID–aID_ = −2 is preferable which is in line with the observation of Chakravarty *et al.*^36^ This is correlated with anion–π interactions between the phosphate backbone and a nucleobase in the GNRA motif (G as a quadrupole, R as an anion) (Fig. 6). Such motifs make up nearly a quarter (22%) of all records found in region A for nucleotides, compared to 9 and 0.2% in regions B and C, respectively. The rest of the records in region A are observed in the other “unpaired” regions of RNA such as internal loops and bulges. GNRA motifs in which “G” represents a quadrupole and “A” represents an anion, are somewhat more common in region C, (5%), but are rare in regions A and B (0.3 and 1%, respectively). Parallelly to our studies, Esmaeeli *et al.* indicated computationally the importance of anion–π interaction in the stabilization of RNA GAAA and GGAG tetraloops within the few selected real systems of living organisms of *E. coli* and *Homo sapiens*, respectively.^69^

### Anion orientation with respect to the ring plane

Due to the low prevalence of linear anions, we consider only planar anion orientations. Anions in ion pairs (*e.g.* acetate bonded to iron in heme) are not analysed in this section. The relative orientation to the quadrupole is defined by the angle between the ring plane and the anion plane. The overall conclusions are that planar anions usually align almost parallel (face–face) to aromatic rings in region A; in region B, the orientation is slightly rotated towards the slanted edge-face; whereas anions located in region C prefer edgewise geometry. This statement is correct for most of the planar anions identified.

The carboxylic groups of ASP and GLU, small carboxylic acids like FMT and ACT, and nitrate anions are noted in particular (see Fig. S14 and S15†). The above observations are also in line with results presented in previous studies where structural analyses and detailed computational analyses of simple anion–π systems revealed that parallel anion orientations are preferred due to overlapping orbitals and their contribution to overall stabilization.^20,21,70–72^ Nevertheless, atypical angle distributions are observed in region A for such ions as citric acid, where no orientation is privileged, and the carboxylic group of heme strongly prefers edgewise geometry.

### Coexisting synthon—ternary interactions

**Fig. 7:**
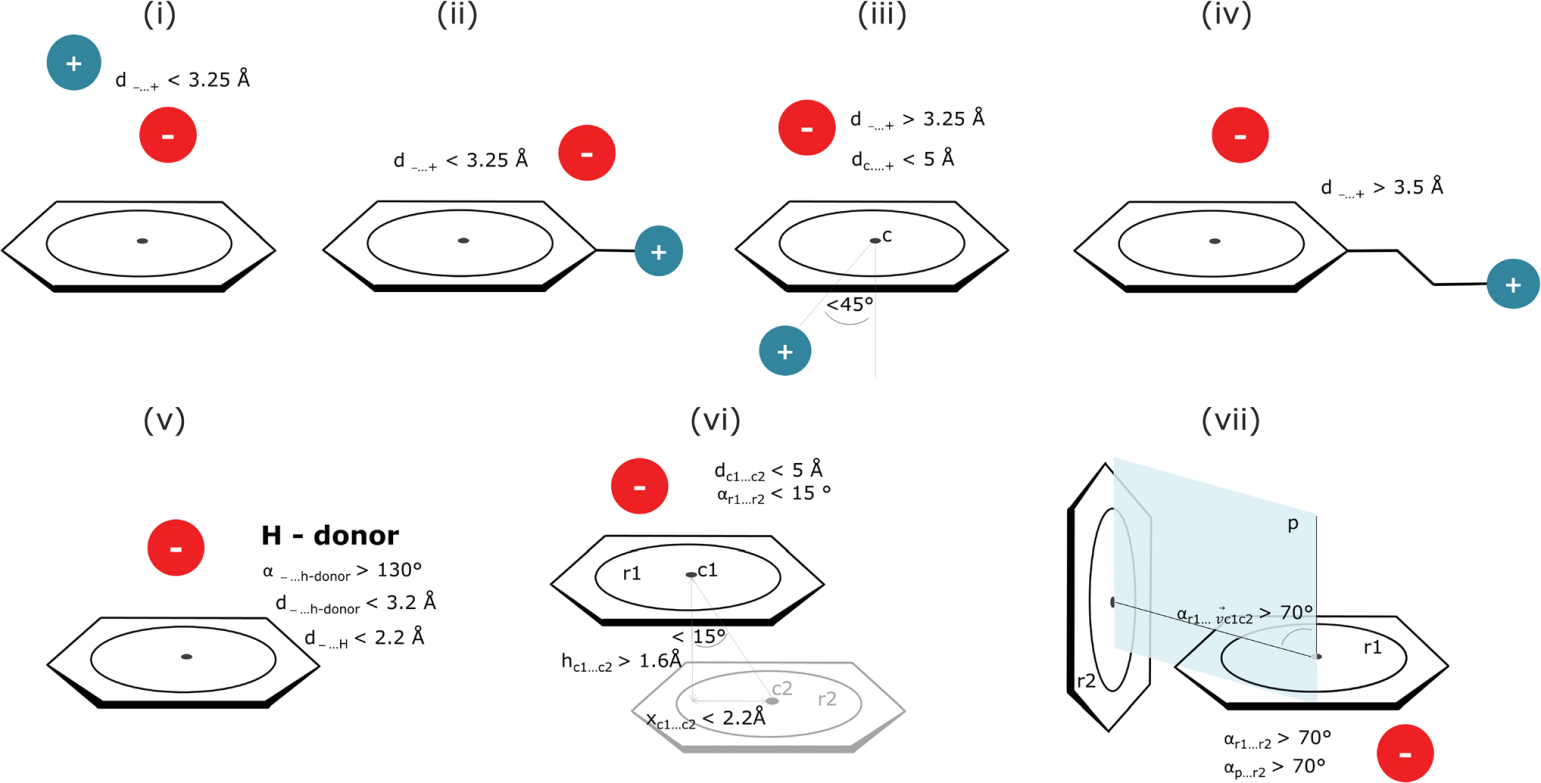
Typical accompanying interactions and the geometrical parameters that we used to distinguish these interactions. (i) Ring⋯anion⋯cation, (ii) anion⋯ring–cation, (iii) anion⋯ring⋯cation, (iv) anion⋯ring- - -cation, (v) ring⋯anion⋯H-donor, (vi) anion⋯ring⋯ring‖, (vii) anion⋯ring⋯ring⊥. Geometric parameters were selected based on the available literature (details in the description below) or determined experimentally.

**Fig. 8:**
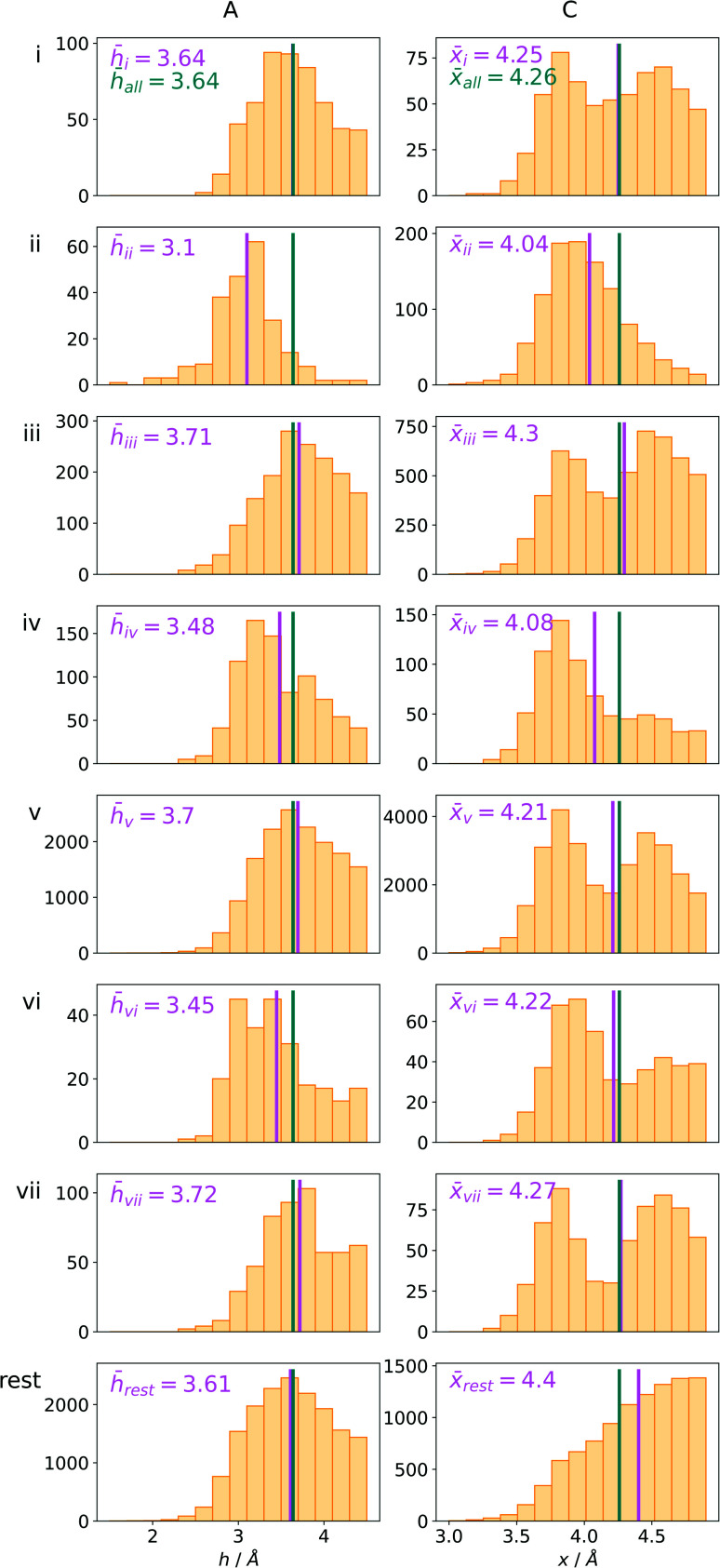
The distributions *h* and densities *x* of anion–arene pairs from identified groups in regions A and C, respectively. The turquoise line represents the mean value for all pairs in the region and the pink line represents the mean value for the relative group.

Bearing in mind the complexity of protein-based systems, we tried to find out which other weak forces accompany anion–aromatic pairs and might affect their stability. We carefully analysed the neighbourhoods around anion–ring pairs and introduced classification of ternary assemblies, as shown in Fig. 7. We distinguish four typical motifs that involve cations: (i) ring⋯anion⋯cation, where the anion and metal cation are on the same site of the ring plane (this condition is not applied to anions in region C) and are close enough for one to suspect that the anion coordinates the metal centre or that electrostatic interaction between ions is dominant; (ii) anion⋯ring–cation, where the cation is coordinated or bonded directly to the aromatic ring and to the anion (the distance to the anion is less than 3.25 Å); (iii) anion⋯ring⋯cation synthons, where the anion and cation are on opposite sides of the ring (this condition is not applied to anions in region C) and the distance between them exceeds 3.25 Å; and (iv) anion⋯ring- - -cation, where the cation is coordinated to a more distant molecular fragment of quadrupole molecule. Moreover, we also considered the (v) ring⋯anion⋯H-donor system, where the anion located above the ring is involved in a strong hydrogen bond, as well as (vi) anion⋯ring⋯ring‖ and (vii) anion⋯ring⋯ring⊥, where another aromatic ring near the anion–π synthon is oriented parallel or perpendicular, respectively, to the primary ring. It is worth noting that sets (i)–(vii) are not disjoint. For example, the structure where the anion is located above the aromatic ring and is involved in interactions with both some metal cation and the hydrogen bond is classified in groups (i) and (v) simultaneously. We also distinguish separately the group where no coexisting interactions are found, hereafter denoted as “(rest)”. The results are summarised in Table 3. The numbers of pairs that belong to each group increase from region A to region C. In each region, the most significant fractions are groups (rest) and (v). The distributions of *h* and *x* over all groups in regions A and C are presented in Fig. 8. The distributions of groups (ii), (iv), and (vi) are especially characteristic, as the mean values of *h* and *x* in the relative groups tend to be smaller than the related mean values for all pairs in the region. Below, we present a brief review of the data from our analysis. Detailed statistics and information are available in the ESI file i_vii_stats.xlsx.†

**Table:** *N*_x_(n) is the number of i–vii (n) type anion–quadrupole pairs found in regions A–C (X). *N*_x_(total) is the number of anion–arene pairs found in the respective regions. Details are provided in i_vii_stats.xlsx

n	A		B		C	
	*N*_A_(n)	*N*_A_(n)/*N*_A_(total)	*N*_B_(n)	*N*_B_(n)/*N*_B_(total)	*N*_C_(n)	*N*_C_(n)/*N*_C_(total)
i	543	1.72	1690	2.11	4239	1.63
ii	227	0.72	1819	2.28	6862	2.64
iii	1618	5.13	4763	5.96	38 659	**14.86**
iv	837	2.65	1446	1.81	4869	1.87
v	15 491	**49.10**	41 249	**51.62**	196 716	**75.61**
vi	245	0.78	506	0.63	3154	1.21
vii	545	1.73	1384	1.73	4535	1.74
Rest	16 489	**52.3**	40 249	**50.4**	69 602	**26.8**
Total	31 551		79 916		260 155	

#### (i) Ring⋯anion⋯cation

To find structures where metal cations are located in the direct neighbourhood of the anion, we prepared a structure set that excludes instances where an aromatic ring has a cation bonded directly to it, the distance between the anion and cation is *d*_−⋯+_ < 3.25 Å, and both ions are on the same side of the ring (for anions in regions A and B). *d*_−⋯+_ was established as an average coordination bond length. It was determined based on literature related to metal biding sites in proteins^73–76^ and magnified to consider strong electrostatic interactions. Only metals were considered as cations, whereas Arg and Lys side chains were investigated in group (v) as hydrogen-bond donors. Iron–sulphur clusters characterised by blurred charges and a wide range of possible oxidation states^77^ were considered as cations only if one of the iron atoms was sufficiently close to the anion. Using this procedure, we find 543 such interactions in region A (1.7%), 1690 in B (2.1%), and 4239 in C (1.6%) (Table 3). The most frequent anions in this set are Asp and Glu (A: 40 and 29.1%, B: 36.2 and 35.3%, and C: 28.7 and 2.1%, respectively), whereas the most frequent cations are Zn^*n*+^ (A: 19.4%, B: 17.7%, C: 23.6%), Mg^2+^ (A: 17.4%, B: 20.5%, C: 17.6%), Mn^*n*+^ (A: 15.5%, B: 11.2%, C: 10.1%), Ca^2+^ (A: 14.8%, B: 12.2%, C: 9.7%), and Fe^*n*+^ (A: 14.1%, B: 19.0%, C: 21.1%) (for more details see the ESI†). The mean *h* and *x* values in this group are almost identical to the average values for all pairs in each region (Fig. 8(i)). Approximately half of the records from group (i) share a part with group (v). This means that the anion interacts with the cation and simultaneously creates a strong hydrogen bond. Moreover we find that a third of the PDB macromolecules contain metal in their structures. The above observation might suggest that the presence of type (i) motifs is not common in macromolecules.

#### (ii) Anion⋯ring–cation

**Fig. 9:**
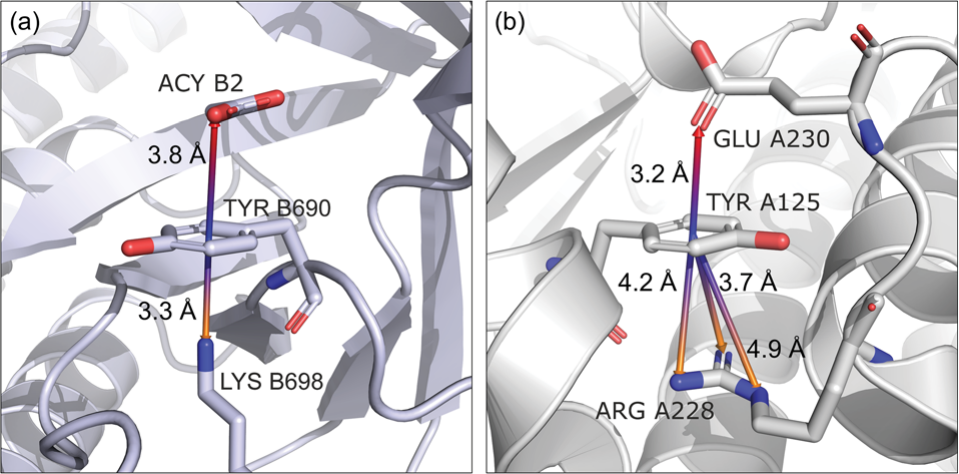
Example of a anion⋯ring⋯cation synthon: fragments of (left) 1VBR^93,94^ and (right) 5OTS^95,96^ structures.

Group (ii) contains structures where *d*_−⋯+_ < 3.25 Å and the metal centre is coordinated directly and simultaneously to aromatic ring. Using these criteria, 227, 1819, and 6862 triads are found in regions A, B, and C, respectively (Table 3). Their contributions are more conspicuous in groups B and C. The vast majority of aromatic rings that coordinate metal cations are histidine (79–96% in A–C), however, porphyrin derivatives such as heme and chlorophyll are also noted (ESI: i_vii_stats.xlsx†). The most common cations in this group are zinc (49–54%), iron (14–17%), and manganese (9–12%). Anions are typically located closer to the ring coordinated to the metal ion. *h̄*_ii_ and *x̄*_ii_ are significantly lower than the mean value for all records found in the respective regions (Fig. 9). This is because of anion–metal coordination rather than stronger anion–π interactions. Over 40% (49% in A) of anions in the identified triads create hydrogen bonds simultaneously (including anion–ring hydrogen bonds).

#### (iii) Anion⋯ring⋯cation

In this group, we search for anion–ring pairs where the quadrupole is engaged in cation–π interactions. As in (i), all rings bonded to metal cations are excluded from this group. Only cations (metal, Arg, or Lys) that are less than 5 Å from the ring's centroid and lie inside the 45 degree spherical sector are considered. Anions and cations are on opposite sides of the ring in the cases of A and B. The fractions of ternary interactions from group (iii) are similar in A and B (5.13 and 5.96%, respectively, which is somewhat larger than reported by Lucas *et al.*^43^), whereas in C almost 15% of anion–ring pairs are accompanied by a cation in this manner. This large difference might be a result of steric effects; if an anion is located in the ring plane (C), it leaves more space for other chemical entities above and below the ring. Approximately 99% of cations located above the ring are cationic side chains of AA. The ratio of Arg to Lys involved in these triads varies from 2.6 : 1 (region A) to 2.9 : 1 (region B and C), which is much more than the ratio of the natural abundance of these molecules (0.9–1.1 to 1)^58^ (which was confirmed in our dataset). The above observation is in general agreement with previous reports, which state that arginine exhibits a stronger tendency to locate above aromatic rings than lysine (see Fig. S17†).^78–81^ The average values *h̄*_iii_ and *x̄*_iii_ in group (iii) are quite close to the average values for each region A, B, and C. This suggests that the cation at the opposite side of the quadrupole does not generally have a large influence on the anion position. This observation does not confirm the canonically acknowledged stabilizing effect of the cation at the reverse side of the quadrupole. However, it might be the result of natural complexity among biological systems. Nevertheless, for RNA *h̄*^RNA^_iii_ is equal to 3.39 Å, which is smaller than the overall average value, in line with the analysis of Lucas *et al.*^43^ A sample image of an anion⋯ring⋯cation synthon is presented in Fig. 9.

#### (iv) Anion⋯ring- - -cation

Interactions are classified to this group if the metal cation is coordinated to an arene (directly or *via* a chain, see Fig. 7), and the distance between the anion and the cation is *d*_−⋯+_ > 3.5 Å (to eliminate metal coordination and ion–ion interactions). It is reasonably assumed that the bonded cation induces polarization and redistribution of the electron density, which enhance the anion–aromatic interactions.

**Fig. 10:**
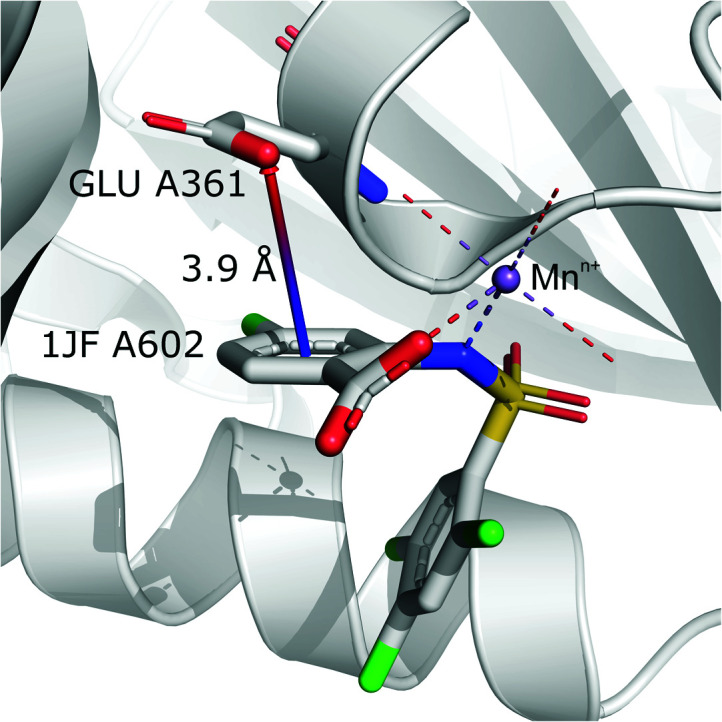
Anion–π interaction in structure 4J04.^97,98^ Coordinated manganese ions presumably induce redistribution of electron density along the ligand molecule, increasing the quadrupole moment of the ring and enhancing anion–π interactions with glutamic acid.

It is worth noting that, although the frequencies of such synthons are not high, they have exclusive importance on aromatic ligands. Almost a third of arenes found in group (iv) are molecules other than AA and NU (in contrast to other groups, which are dominated by AA or NU; see Table 4 and i_vii_stats.xlsx for more details†). This trend is observed mostly in A (29%), whereas in C just histidine makes up 76% of aromatics. Based on the above, we assume that anion–π interactions that are assisted by cation-induced electron density relocation have special importance when bonding external ligands to macromolecules (all ligand codes from this group are available in the ESI†). The mean values of *h̄*_iv_ and *x̄*_iv_ are significantly lower than values for all records found in the respective regions (Fig. 8). This is presumed to be an effect of anion–π interaction reinforcement. Typical cations that coordinate aromatic molecules within this class are zinc (34–39%), magnesium (13–24%), and iron (12–14%). One example of such a synthon is structure 4J04 presented in Fig. 10.

**Table:** Fractions of various aromatic ring types in (iv)

Ring type	A		B		C	
	Count	%	Count	%	Count	%
RNA	115	13.74	109	7.54	318	6.53
DNA	8	0.96	26	1.80	21	0.43
Protein	471	56.27	1000	69.16	3957	81.27
Ligand	243	29.03	311	21.51	573	11.77

#### (v) Ring⋯anion⋯H-donor

**Fig. 11:**
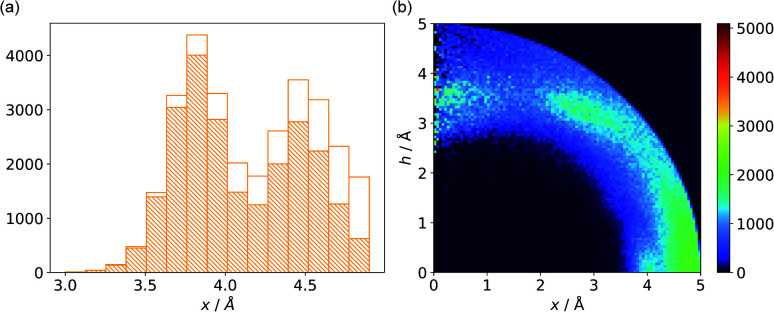
(a) Distribution of *x* values for anions from region C. The dashed bars represent the number of anions that create hydrogen bonds with aromatic rings, whereas the white bars represent all hydrogen bonds found in subsequent ranges. (b) Anion density around aromatic rings after exclusion of pairs with anion–ring hydrogen bonds.

In cases where hydrogen atoms were present in the model (mainly from hybrid solutions like X-ray + NMR, *etc.*) we used the current protonation state of the whole molecule. If H atoms were not present in the PDB file we added them only to amino acid residues (so in such cases, ligands were not considered as H-bond donors). For more details see ESI.† Only anions were considered as H-bond acceptors (including ligands). The parameters for detection of hydrogen bonds were set as follows: angle acceptor–hydrogen-donor *α*_−⋯h-donor_ > 130°, distance acceptor–donor *d*_−⋯h-donor_ < 3.2 Å and acceptor–hydrogen distance *d*_−⋯h-donor_ < 2.2 Å (Fig. 7). (v) is the most abundant group; approximately 50% of anions in A and B and over 75% of anions in C create hydrogen bonds. In C, the hydrogen-bond donor and quadrupole are the same molecule in the vast majority (78%) of cases. This confirms our initial statement that the two maxima apparent in the *x* and *h* histograms (Fig. 2a) are related to hydrogen bonds with histidine and tryptophan (*x* ∼ 3.8 Å) or tyrosine (*x* ∼ 4.5 Å) (compare with Fig. 11a). Therefore, we decided to create 2D histograms of anion density around the aromatic ring after excluding anion–ring pairs that form hydrogen bonds (see Fig. 11b). Regions A and B are thus even more distinguishable. It should be stressed that the number of accompanying hydrogen bonds is approximately 1.5 times greater than the number of quadrupole–anion pairs found. This is due to the fact that one anion creates a strong hydrogen bond with more than one hydrogen donor relatively often.

#### (vi) Anion⋯ring⋯ring‖

This motif represents anion–ring pairs, where a primary ring (quadrupole) is engaged in π–π interactions with another aromatic ring. To classify a pair of aromatic rings as a parallel pair we require that the angle between the two rings (between their normal vectors) is *α*_r1⋯r2_ < 15°, the distance between their centroids is *d*_c1⋯c2_ < 5 Å, *h*_c1⋯c2_ > 1.6 Å, and *x*_c1⋯c2_ < 2.2 Å (Fig. 7).

The last restriction is that the additional ring centroid should be inside the 15° spherical sector of the quadrupole. A feature unique to the (vi) group is a high fraction of ribonucleotides that act as quadrupoles (see Table 5 and i_vii_stats.xlsx†). In the case of region A, over 60% of central aromatic rings are RNA NU (G represents almost 25%, U represents 17.5%, and A represents 11%).

**Table:** Fractions of specific aromatic ring types in vi

Ring type	A		B		C	
	Count	%	Count	%	Count	%
RNA	149	60.82	93	18.38	618	19.59
DNA	5	2.04	40	7.91	119	3.77
Protein	66	26.94	288	56.92	1690	53.58
Ligand	25	10.20	85	16.80	727	23.05

#### (vii) Anion⋯ring⋯ring⊥

**Fig. 12:**
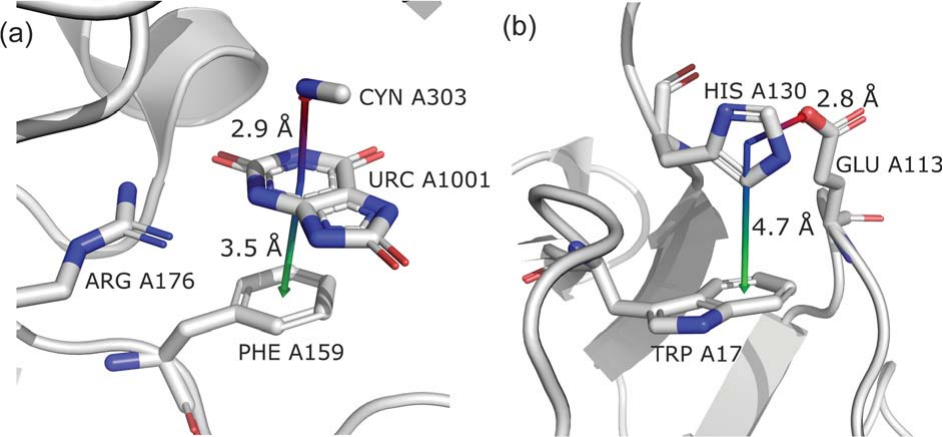
Examples of anion–quadrupole pair triads: (a) 3BJP^99,100^ and (b) 3VPY^101,102^ structures.

In this motif, another aromatic ring in the neighbourhood of the anion–π synthon is oriented perpendicular to the quadrupole ring. Only pairs of quadrupoles, the centroids of which are closer than 5 Å, are considered. The angle between the normal vectors of ring planes is restricted to *α*_r1⋯r2_ > 70°. The angle between the normal vector of the quadrupole and the vector that connects the centroids of the two rings is *α*_r1⋯rc1c2_ > 70° (Fig. 7). The angle between the second ring plane and the plane defined by the vector connecting the centroids, and the normal vector of the primary ring is *α*_p⋯r2_ > 70°. Over 94% of central aromatic rings in this group are AA. In general, the group of perpendicular π–π synthons is much more numerous than (vi) in all regions. This observation is in agreement with the general tendency of small, aromatic rings to form edge-face (T-shaped) pairs.^82^ Moreover, our results are in agreement with those presented by Lucas *et al.*,^43^ despite the differences in methodology. Analysis of mutual π–π distributions (Fig. S18†) shows that the maximum of ring occurrence in the plane appears when *x* is approximately 5 Å. This suggests that sufficient analysis of group (vii) would require a search in a larger sphere. Examples of synthons (vi) and (vii) are presented in Fig. 12.

#### Rest

This group is a result of the exclusion of groups (i)–(vii) and contains only anion–ring pairs that are not engaged in any of the synthons described above. A summary of this group is presented in Table 6. In general, the statistics for this group are similar to those for all anion–ring pairs in regions A–C. In the case of A, the shape of the *h* value distribution differs from those obtained for all anion–ring pairs in region A (see Fig. S6†), although *h̄*_rest_ is almost the same. In the case of C, an *x* value distribution with a completely different shape is observed. This confirms that two strong maxima on the 2D histogram can be assigned to the presence of hydrogen bonds.

**Table:** The fraction of each type of aromatic ring in group rest

Ring type	A		B		C	
	Count	%	Count	%	Count	%
RNA	894	5.42	930	2.31	2236	3.21
DNA	69	0.42	180	0.45	428	0.61
Protein	14 572	88.37	37 667	93.58	62 843	90.29
Ligand	954	5.79	1472	3.66	4095	5.88

### Aromatics and other chemical entities

**Fig. 13:**
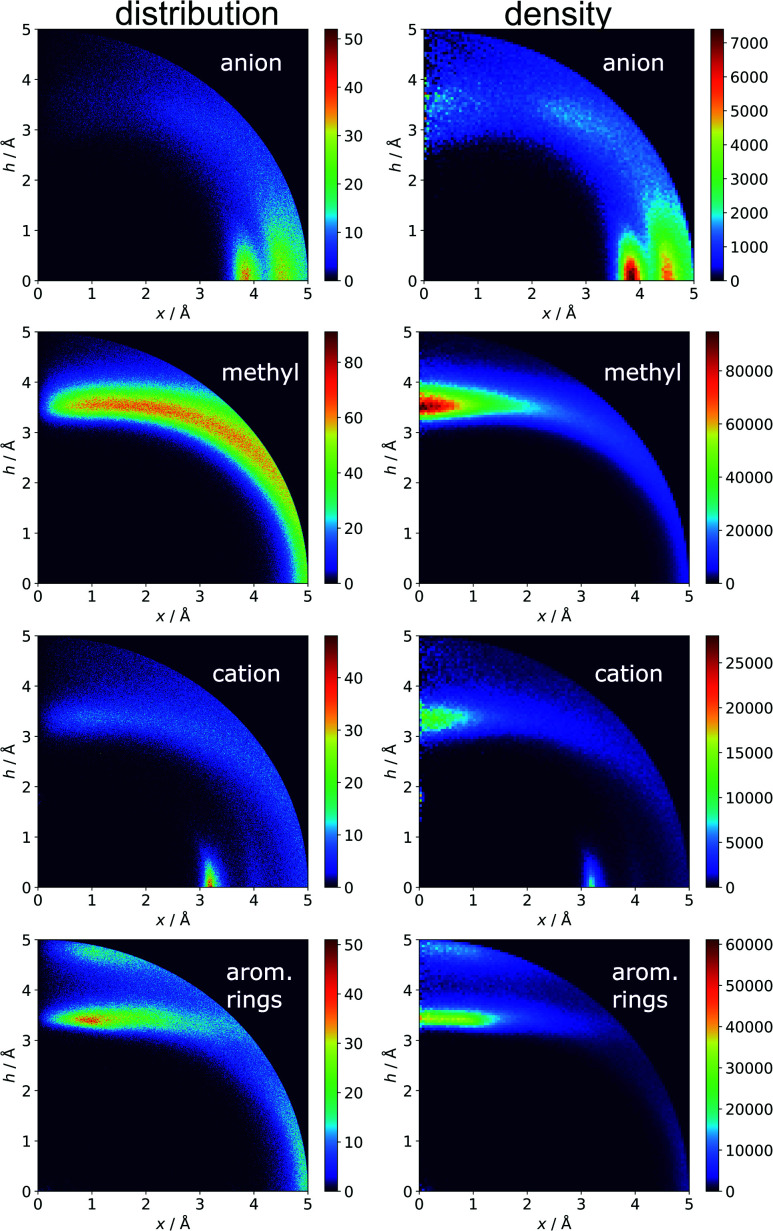
Comparison of the distributions (bin size: Δ*x* = Δ*h* = 0.01 Å) and densities (bin size: Δ*x* = Δ*h* = 0.05 Å) of anions, methyl groups, cations, and other aromatic rings around aromatic rings. The presented histograms are calculated for unique pairs in the non-redundant set of macromolecules with resolution *r* ≤ 2.5 Å.

In the final section of this work, we present the results of searches for other chemical entities around aromatic rings. Fig. 13 shows the juxtaposition of the results. First, we consider methyl groups from alanine, isoleucine, leucine, threonine, and valine side chains. These aggregate above the arene (*h* in the range 3–4 Å). This trend is similar for aromatic AAs, NUs, and ligands (Fig. S16†). We also analyse cation distributions. Metal ions locate preferentially in the plane of the ring or in other parts of the sphere, and avoid the area above the ring. In contrast, the cationic side chains of Arg, and Lys (Fig. S17†) avoid positions in the ring plane. Geometrical preferences differ significantly among AAs, *i.e.* arginine is more condensed above the ring, while lysine tends to diffuse across the entire sphere. This might suggest that the electronic structure of the cationic group and orbital contributions also affect cation–π interaction strengths. Studies have been conducted that consider basic AAs from the standpoint of their ability to form cation–pi interactions.^78–80^ However, the authors of these works used different methodologies. In particular the spheres searched had larger radii. Nevertheless, our results confirm that arginine exhibits a greater tendency to locate above the aromatic ring than lysine. On the other hand, despite well documented metal–π interactions,^83–86^ most metal cations locate preferentially in the ring plane instead of above it. Finally, other aromatics mostly locate above arene with two maxima; the first appears when *h* is in the 3–4 Å range, and the second appears when *h* is approximately 5 Å (the histograms show the locations of the ring centroids). Finally, we analyse distribution of other aromatic rings around quadrupoles in 5 Å sphere. In the case of AAs quadrupoles, other rings locate preferentially above (*h* approximately 5 Å) or in the plane (*x* approximately 5 Å) of quadrupole. This corresponds to a T-shaped and distant π–π interaction.^82^ In the case of NU and ligands, strong π–π interaction is preferred (maximum in *h* approximately 3.5 Å, see Fig. S18†). This comparison allows the following conclusion to be drawn: anion–π interactions among macromolecules are much less common than other supramolecular interactions with aromatic rings, so it is likely that their importance to biomolecular stability is statistically lower than importance of other supramolecular forces. However, as shown in the previous sections, they might be crucial to ligand binding in adducts that involve ring systems with positive quadrupole moments and notable polarizability. Moreover, this basic comparison suggests that it is worth performing a similar analysis with the use (*x*, *h*) coordinate system for other non-covalent interactions consisting of aromatic rings. As we have shown, the choice of the coordinate system is extremely important for the proper reproduction of statistical space occupation by the interacting species. Therefore such analysis would be of great importance to validation of information obtained from the analysis using (*R*, *α*) coordinate system.

## Conclusions

Anion–aromatic ring interactions in macromolecules were explored comprehensively *via* consideration of all non-redundant PDB records including amino acids, nucleobases, and, non-standard residues never analysed before. The results provided a substantial update that covers various binary and ternary interaction motifs. Unlike previous studies of anion–π interactions in macromolecules, we decided to use the (*x*, *h*) coordinate system which, in our opinion, (i) allows one to distinguish more effectively the space regions with increased motif densities, and (ii) is more intuitive to interpret. To the best of our knowledge, this approach has never been used to describe anion–aromatic interactions, although it was used several times in cation–π studies.^81,87^ Based on a 2D map of anion distributions and densities around aromatic rings, we defined and carefully analysed three cylinders, or cylinder-shell space fragments, covering the space above the ring centroid (A), above the ring bonds (B), and in the peripheral area close to the ring plane (C). This approach let us visualise for the first time local compaction of anions above the aromatic ring, whereas in previous works, monotonic growth in anion occurrence with distance from the centroid was presented.^43^ Moreover, ratio of non-standard (ligands, cofactors, *etc.*) to standard (AA, NU) residues increase from region C to region A. This suggests that anion–π interactions are probably important to docking of the ligand and might be essential from a drug design perspective. From sequence and secondary structure analyses, we found that anion–π interactions might also influence GNRA tetraloop thermostability in RNA and on helical structures in proteins. The ratio of Asp and Glu anionic residues located above the aromatic ring to those located within 5 Å of its centre suggests that the nature of such interactions is similar for both of these residues. Planar anions located above the aromatic ring, prefer positions that are rotated slightly from being strictly parallel to the quadrupole and moved slightly from being just above the centre of a ring. In contrast, those located in the plane of the ring prefer edgewise interactions. Analysis of ternary interaction motifs shows that the most numerous moieties (∼50% in A and B, and ∼75% in C) are anion–ring pairs where the anion forms a hydrogen bond simultaneously. The most interesting ternary interactions are anion–ring pairs, where the cation is coordinated by the quadrupole's chain. In this group, a surprisingly large portion of the quadrupole are ligands. A comparative analysis of the distributions and densities of anions and other moieties indicates that anion–π interactions should be essential to specific situations that involve rings with positive quadrupole moments, although a number of relevant motifs are less common than other interactions that involve aromatic rings.

To summarize, the results of our updated study performed using an alternative model significantly expand previous analyses of interactions involving aromatic AAs in proteins.^38,43,63^ The novelty we bring to the discussion of anion–pi interaction in biological systems involve: (i) analysis of all possible aromatic residues, (ii) critical discussion on the implications born by a choice of coordination system, (iii) proof for local anion compaction above the aromatic rings as the example of the new feature definitely hidden for the standard model used previously, (iv) extended sequence analysis of RNA strands, (v) clear distinction of anion–π and anion–ring interactions possible within one PDB search protocol, and (vi) serious extension of coexisting interactions in ternary systems. Moreover, as a result of our studies we share our original searching tool which can be used by other researchers in more efficient, future PDB mining.

## Data availability

Processed data are available in the xlsx file in the ESI.† Any set of structures in mmCIF format can be processed with code available at https://github.com/chemiczny/PDB_supramolecular_search.

## Author contributions

E. K.-G.: conceptualization, investigation, methodology design, data curation, formal analysis, validation, writing and visualisation of main text and ESI,† implementation of supporting algorithms, data curation. M. G.: investigation, programming, software development; designing computer programs; implementation of the computer code and supporting algorithms; testing of existing code components, formal analysis, co-authored the methodology, edited text of manuscript and ESI,† data curation. R. K., A. J. B. and R. P.: preliminary conceptualization, supervision, developed discussion, writing – review & editing.

## Conflicts of interest

There are no conflicts to declare.

## Supplementary Material

SC-013-D2SC00763K-s001

SC-013-D2SC00763K-s002

## References

[cit1] Zhao Y., Beuchat C., Domoto Y., Gajewy J., Wilson A., Mareda J., Sakai N., Matile S. (2014). J. Am. Chem. Soc..

[cit2] Bornhof A.-B. B., Bauzá A., Aster A., Pupier M., Frontera A., Vauthey E., Sakai N., Matile S. (2018). J. Am. Chem. Soc..

[cit3] López-Andarias J., Bauzá A., Sakai N., Frontera A., Matile S. (2018). Angew. Chem., Int. Ed..

[cit4] Shahraki A., Ebrahimi A., Rezazadeh S., Behazin R. (2021). Mol. Syst. Des. Eng..

[cit5] Kepler S., Zeller M., Rosokha S. V. (2019). J. Am. Chem. Soc..

[cit6] Liao J.-Z., Meng L., Jia J.-H., Liang D., Chen X.-L., Yu R.-M., Kuang X.-F., Lu C.-Z. (2018). Chem.–Eur. J..

[cit7] You M. H., Di Y. M., Li M. H., Li H. H., Lin M. J. (2020). Dyes Pigm..

[cit8] Hao P., Zhu H., Pang Y., Shen J., Fu Y. (2020). Cryst. Growth Des..

[cit9] Jankowski R., Zakrzewski J. J., Surma O., Ohkoshi S., Chorazy S., Sieklucka B. (2019). Inorg. Chem. Front..

[cit10] Guha S., Saha S. (2010). J. Am. Chem. Soc..

[cit11] Chifotides H. T., Schottel B. L., Dunbar K. R. (2010). Angew. Chem., Int. Ed..

[cit12] Wang D.-X., Wang M.-X. (2013). J. Am. Chem. Soc..

[cit13] Savastano M., Bazzicalupi C., Giorgi C., García-Gallarín C., de la Torre M. D. L., Pichierri F., Bianchi A., Melguizo M. (2016). Inorg. Chem..

[cit14] Mareda J., Matile S. (2009). Chem.–Eur. J..

[cit15] Adriaenssens L., Estarellas C., Vargas Jentzsch A., Martinez Belmonte M., Matile S., Ballester P. (2013). J. Am. Chem. Soc..

[cit16] Roy A., Saha D., Mandal P. S., Mukherjee A., Talukdar P. (2017). Chem.–Eur. J..

[cit17] Huang W. L., Wang X. D., Ao Y. F., Wang Q. Q., Wang D. X. (2020). J. Am. Chem. Soc..

[cit18] Chifotides H. T., Giles I. D., Dunbar K. R. (2013). J. Am. Chem. Soc..

[cit19] Arranz-Mascarós P., Bazzicalupi C., Bianchi A., Giorgi C., Godino-Salido M.-L. L., Gutiérrez-Valero M.-D. D., Lopez-Garzón R., Savastano M. (2013). J. Am. Chem. Soc..

[cit20] Kuzniak E., Hooper J., Srebro-Hooper M., Kobylarczyk J., Dziurka M., Musielak B., Pinkowicz D., Raya J., Ferlay S., Podgajny R. (2020). Inorg. Chem. Front..

[cit21] Kuzniak-Glanowska E., Glosz D., Niedzielski G., Kobylarczyk J., Srebro-Hooper M., Hooper J. G. M., Podgajny R. (2021). Dalton Trans..

[cit22] Liao J.-Z., Zhang H.-L., Wang S.-S., Yong J.-P., Wu X.-Y., Yu R., Lu C.-Z. (2015). Inorg. Chem..

[cit23] Baldoví J. J., Coronado E., Gaita-Ariño A., Gamer C., Giménez-Marqués M., Mínguez Espallargas G. (2014). Chem.–Eur. J..

[cit24] Eytel L. M., Fargher H. A., Haley M. M., Johnson D. W. (2019). Chem. Commun..

[cit25] Cotelle Y., Lebrun V., Sakai N., Ward T. R., Matile S. (2016). ACS Cent. Sci..

[cit26] Schwans J. P., Sunden F., Lassila J. K., Gonzalez A., Tsai Y., Herschlag D. (2013). Proc. Natl. Acad. Sci. U. S. A..

[cit27] Estarellas C., Frontera A., Quiñonero D., Deyà P. M. (2011). Angew. Chem., Int. Ed..

[cit28] Zlatović M. V., Borozan S. Z., Nikolić M. R., Stojanović S. Đ. (2015). RSC Adv..

[cit29] Estarellas C., Frontera A., Quiñonero D., Deyà P. M. (2011). Chem.–Asian J..

[cit30] Bauzá A., Quiñonero D., Deyà P. M., Frontera A. (2014). Chem.–Eur. J..

[cit31] Ellenbarger J. F., Krieger I. V., Huang H. L., Gómez-Coca S., Ioerger T. R., Sacchettini J. C., Wheeler S. E., Dunbar K. R. (2018). J. Chem. Inf. Model..

[cit32] Ribić V. R., Stojanović S. Đ., Zlatović M. V. (2018). Int. J. Biol. Macromol..

[cit33] Smith M. S., Lawrence E. E. K., Billings W. M., Larsen K. S., Bécar N. A., Price J. L. (2017). ACS Chem. Biol..

[cit34] Breberina L. M., Milčić M. K., Nikolić M. R., Stojanović S. D. (2015). J. Biol. Inorg Chem..

[cit35] Kapoor K., Duff M. R., Upadhyay A., Bucci J. C., Saxton A. M., Hinde R. J., Howell E. E., Baudry J. (2016). Biochemistry.

[cit36] Chakravarty S., Sheng Z. Z., Iverson B., Moore B. (2012). FEBS Lett..

[cit37] Mbaye M. N., Hou Q., Basu S., Teheux F., Pucci F., Rooman M. (2019). Sci. Rep..

[cit38] Chakravarty S., Ung A. R., Moore B., Shore J., Alshamrani M. (2018). Biochemistry.

[cit39] Jackson M. R., Beahm R., Duvvuru S., Narasimhan C., Wu J., Wang H. N., Philip V. M., Hinde R. J., Howell E. E. (2007). J. Phys. Chem. B.

[cit40] Philip V., Harris J., Adams R., Nguyen D., Spiers J., Baudry J., Howell E. E., Hinde R. J. (2011). Biochemistry.

[cit41] Bauzá A., Quiñonero D., Deyà P. M., Frontera A. (2013). Chem.–Asian J..

[cit42] Yurenko Y. P., Bazzi S., Marek R., Kozelka J. (2017). Chem.–Eur. J..

[cit43] Lucas X., Bauzá A., Frontera A., Quiñonero D. (2016). Chem. Sci..

[cit44] Jenkins D. D., Harris J. B., Howell E. E., Hinde R. J., Baudry J. (2013). J. Comput. Chem..

[cit45] Mahadevi A. S., Sastry G. N. (2016). Chem. Rev..

[cit46] Berman H. M. (2000). Nucleic Acids Res..

[cit47] Berman H., Henrick K., Nakamura H. (2003). Nat. Struct. Mol. Biol..

[cit48] Burley S. K., Bhikadiya C., Bi C., Bittrich S., Chen L., Crichlow G. V., Christie C. H., Dalenberg K., Di Costanzo L., Duarte J. M., Dutta S., Feng Z., Ganesan S., Goodsell D. S., Ghosh S., Green R. K., Guranović V., Guzenko D., Hudson B. P., Lawson C. L., Liang Y., Lowe R., Namkoong H., Peisach E., Persikova I., Randle C., Rose A., Rose Y., Sali A., Segura J., Sekharan M., Shao C., Tao Y.-P., Voigt M., Westbrook J. D., Young J. Y., Zardecki C., Zhuravleva M. (2021). Nucleic Acids Res..

[cit49] Glanowski M. and Kuzniak-Glanowska E., 2021, https://github.com/chemiczny/PDB_supramolecular_search

[cit50] Hamelryck T., Manderick B. (2003). Bioinformatics.

[cit51] Cock P. J. A., Antao T., Chang J. T., Chapman B. A., Cox C. J., Dalke A., Friedberg I., Hamelryck T., Kauff F., Wilczynski B., de Hoon M. J. L. (2009). Bioinformatics.

[cit52] Li W., Godzik A. (2006). Bioinformatics.

[cit53] Fu L., Niu B., Zhu Z., Wu S., Li W. (2012). Bioinformatics.

[cit54] The pandas development team , pandas-dev/pandas: Pandas, Zenodo, 2020

[cit55] McKinney W. , in Proceedings of the 9th Python in Science Conference, 2010, pp. 56–61

[cit56] Hunter J. D. (2007). Comput. Sci. Eng..

[cit57] Schrödinger L. , The PyMOL Molecular Graphics System, Version 2.1.1, Schrödinger, LLC, 2015

[cit58] Kozlowski L. P. (2017). Nucleic Acids Res..

[cit59] Mignon P., Loverix S., Steyaert J., Geerlings P. (2005). Nucleic Acids Res..

[cit60] Ferreira de Freitas R., Schapira M. (2017). MedChemComm.

[cit61] Kabsch W., Sander C. (1983). Biopolymers.

[cit62] Touw W. G., Baakman C., Black J., te Beek T. A. H., Krieger E., Joosten R. P., Vriend G. (2015). Nucleic Acids Res..

[cit63] Chakrabarti P., Bhattacharyya R. (2007). Prog. Biophys. Mol. Biol..

[cit64] Waters M. L. (2004). Biopolymers.

[cit65] Chastain M., Tinoco I. (1991). Prog. Nucleic Acid Res. Mol. Biol..

[cit66] Batey R. T., Rambo R. P., Doudna J. A. (1999). Angew. Chem., Int. Ed..

[cit67] Cheong C. and Cheong H.-K., RNA Structure: Tetraloops, in Encyclopedia of Life Sciences, John Wiley & Sons, Ltd, Chichester, UK, 2010

[cit68] Fiore J. L., Nesbitt D. J. (2013). Q. Rev. Biophys..

[cit69] Esmaeeli R., Piña M. d. l. N., Frontera A., Pérez A., Bauzá A. (2021). J. Chem. Theory Comput..

[cit70] Estarellas C., Quiñonero D., Deyà P. M., Frontera A. (2013). ChemPhysChem.

[cit71] Kuzniak E., Pinkowicz D., Hooper J., Srebro-Hooper M., Hetmańczyk Ł., Podgajny R. (2018). Chem.–Eur. J..

[cit72] Wilson J., Maxson T., Wright I., Zeller M., Rosokha S. V. (2020). Dalton Trans..

[cit73] Harding M. M. (2006). Acta Crystallogr., Sect. D: Biol. Crystallogr..

[cit74] Lev B. , Roux B. and Noskov S. Y., Encycl. Met., 2013, pp. 2112–2118

[cit75] Durdagi S. , Roux B. and Noskov S. Y., Encycl. Met., 2013, pp. 1809–1815

[cit76] Laitaoja M., Valjakka J., Jänis J. (2013). Inorg. Chem..

[cit77] Johnson D. C., Dean D. R., Smith A. D., Johnson M. K. (2005). Annu. Rev. Biochem..

[cit78] Gallivan J. P., Dougherty D. A. (1999). Proc. Natl. Acad. Sci. U. S. A..

[cit79] Chakkaravarthi S., Gromiha M. M. (2006). Polymer.

[cit80] Gromiha M. M., Santhosh C., Ahmad S. (2004). Int. J. Biol. Macromol..

[cit81] Kumar K., Woo S. M., Siu T., Cortopassi W. A., Duarte F., Paton R. S. (2018). Chem. Sci..

[cit82] Thakuria R., Nath N. K., Saha B. K. (2019). Cryst. Growth Des..

[cit83] Hagiwara Y., Matsumura H., Tateno M. (2009). J. Am. Chem. Soc..

[cit84] Santarelli V. P., Eastwood A. L., Dougherty D. A., Ahern C. A., Horn R. (2007). Biophys. J..

[cit85] Xue Y., Davis A. V., Balakrishnan G., Stasser J. P., Staehlin B. M., Focia P., Spiro T. G., Penner-Hahn J. E., O'Halloran T. V. (2008). Nat. Chem. Biol..

[cit86] Gokel G. W., Barbour L. J., Ferdani R., Hu J. (2002). Acc. Chem. Res..

[cit87] Reddy A. S., Sastry G. M., Sastry G. N. (2007). Proteins: Struct., Funct., Bioinf..

[cit88] Wu R. , Clancy S., Joachimiak A. and MCSG, The crystal structure of sigma-54-dependent transcriptional regulator domain from *Chlorobium tepidum* TLS, 2009, DOI: 10.2210/pdb3K2N/pdb

[cit89] Cavalier M. C. , Kim S. G., Neau D. and Lee Y. H., PFKFB3 in complex with PPi, 2011, DOI: 10.2210/pdb3QPU/pdb

[cit90] Cavalier M. C., Kim S.-G., Neau D., Lee Y.-H. (2012). Proteins: Struct., Funct., Bioinf..

[cit91] Park H. , Lohman J. and Disney M. D., Myotonic Dystrophy Type 2 RNA: Structural Studies and Designed Small Molecules that Modulate RNA Function, 2013, DOI: 10.2210/pdb4K27/pdb

[cit92] Childs-Disney J. L., Yildirim I., Park H., Lohman J. R., Guan L., Tran T., Sarkar P., Schatz G. C., Disney M. D. (2014). ACS Chem. Biol..

[cit93] Ihsanawati T. Kumasaka , Kaneko T., Nakamura S. and Tanaka N., Crystal structure of complex xylanase 10B from *Thermotoga maritima* with xylobiose, 2004, DOI: 10.2210/pdb1VBR/pdb

[cit94] Ihsanawati, Kumasaka T., Kaneko T., Morokuma C., Yatsunami R., Sato T., Nakamura S., Tanaka N. (2005). Proteins: Struct., Funct., Bioinf..

[cit95] Brear P. , De Fusco C., Iegre J., Yoshida M., Mitchell S., Rossmann M., Carro L., Sore H., Hyvonen M. and Spring D., The crystal structure of CK2alpha in complex with an analogue of compound 22, 2017, DOI: 10.2210/pdb5OTS/pdb

[cit96] Iegre J., Brear P., De Fusco C., Yoshida M., Mitchell S. L., Rossmann M., Carro L., Sore H. F., Hyvönen M., Spring D. R. (2018). Chem. Sci..

[cit97] Coulombe R. , Crystal structure of hcv ns5b polymerase in complex with 4-chloro-2-{[(2,4,5-trichlorophenyl)sulfonyl]amino}benzoic acid, 2013, DOI: 10.2210/pdb4J04/pdb

[cit98] Stammers T. A., Coulombe R., Rancourt J., Thavonekham B., Fazal G., Goulet S., Jakalian A., Wernic D., Tsantrizos Y., Poupart M. A., Bös M., McKercher G., Thauvette L., Kukolj G., Beaulieu P. L. (2013). Bioorg. Med. Chem. Lett..

[cit99] Gabison L. , Prange T., Colloc'h N., El Hajji M., Castro B. and Chiadmi M., Urate oxidase cyanide uric acid ternary complex, 2007, DOI: 10.2210/pdb3BJP/pdb

[cit100] Gabison L., Prangé T., Colloc'h N., El Hajji M., Castro B., Chiadmi M. (2008). BMC Struct. Biol..

[cit101] Yuan Y. A. and Machida S., Crystal structure of *Arabidopsis* DDL FHA domain, 2012, DOI: 10.2210/pdb3VPY/pdb

[cit102] Machida S., Yuan Y. A. (2013). Mol. Plant.

