# Supplementary material for: Mining anion–aromatic interactions in the Protein Data Bank
Source: Chem Sci. 2022 Mar 1;13(14):3984–98. doi: 10.1039/d2sc00763k (PMC8985504; doi:10.1039/d2sc00763k)
Supplement: SC-013-D2SC00763K-s002 [file SC-013-D2SC00763K-s002.pdf]

Electronic Supplementary Material (ESI) for Chemical Science.
This journal is © The Royal Society of Chemistry 2022

Electronic Supporting Information (ESI)

Mining anion-aromatic interactions in the Protein Data Bank

Emilia Kuzniak-Glanowska[a], Michał Glanowski[b], Rafał Kurczab[c], Andrzej J. Bojarski[c,*], Robert Podgajny[a,*]

[a] *Faculty of Chemistry, Jagiellonian University, Gronostajowa 2, 30-387 Kraków, Poland*

[b] *Jerzy Haber Institute of Catalysis and Surface Chemistry, Polish Academy of Sciences, Niezapominajek 8, 30-239 Kraków, Poland*

[c] *Maj Institute of Pharmacology, Polish Academy of Sciences, Smętna 12, 31-343 Kraków, Poland*

Table of content

Methodology details 2
- Unique records 2
- Aromatic ring detection 2
- Anions classification 3
- Cations classification 4
- Neighbouring ring 4
- Hydrogen bonds 4
- Differences between coordinate systems 5
  - *R* and *α* coordinate system (*R*,*α*) 5
  - *h* and *x* coordinate system (*x*,*h*) 6

Additional results 9
- Histogram for all structures from PDB 9
- Occurrences of anion-ring pairs - summary 10
- Anions distribution for the most common residues 15
- Structure type analysis 17
- Sequence analysis 18
- Planar anions orientation 23
- Aromatics and other chemical entity around rings 25

Methodology details

Almost all of 171588 structural cif files from Protein Data Bank[1–3] (access 29.11.2020; 6 of them were unparseable using Biopython) were analysed with our Python program (public access: https://github.com/chemiczny/PDB_supramolecular_search; used with Python 3.6 version). Runtime to process all PDB structures is around 4 hours using 18 nodes with 24 processors each. For reading and parsing files, a PDB submodule[4] from Biopython package[5] was used. Irrespective of origin (X-ray, NMR, electron microscopy, etc.), each model was treated by the same procedure. In the case of multiple geometry files (mostly NMR solutions) each model (frame) was parsed independently. Only an „a" solution from disordered structures was processed. Due to the wrong type assignment in plenty of mmcif files (mostly qualified as protein even in case of lack of amino acid), we implemented a simple method based on macromolecule's residues content, for classification structural model as protein, DNA, RNA, DNA-RNA, protein-DNA, protein-RNA or protein-DNA-RNA. We didn't include self-interaction (when anion and aromatic ring are in the same molecule).

Unique records

This step was done using the CD-HIT program.[6,7] In the case of sequences shorter than 16 residues, chains were classified as one cluster if they were identical. For longer chains, we required 95% sequence identity, and that alignment covers at least 90% of the chain. Using data about clusters we introduce the "sequence id" attribute to each residue occurring in obtained records. Finally, we saved only a unique anion-aromatic ring pair consisting of ring name, sequence id, and residue number for aromatics as well as on anion name, sequence id, residue number, and anion group number for anions. The anion group number was also introduced by us, to distinguish different COO^- groups of polycarboxylic molecules and treat them as separate individuals. By using such a procedure we count redundant interactions in different chains of one structure only once.

Aromatic ring detection

Every single residue was analysed as potential π-acid. Our procedure used graph representation of molecule (we treat atoms as nodes and bonds as edges). To construct such data structure we analysed distances between all atoms from residue. Hydrogen atoms were omitted in this step. We used 120% of the sum of atoms' valence radius value determined for single bonds as a threshold for bond lengths.[8] We took into consideration only 5 and 6 membered rings consisting

of carbon, nitrogen and oxygen. To classify a set of atoms as a ring we used only information about graph structure. We did not assume that an atom can be the part of a ring based on its name in a cif file. For this purpose, networkx implementation[9] of algorithm for finding a fundamental set of cycles was used.[10] To determine if a ring is aromatic we tested its flatness. During this step we analysed both atoms from the cycle and atoms of substituents (marked as R in the Figure S 1) connected directly to the ring. An average normal vector (v) of the ring plane was calculated based on cross products of vectors along every two subsequent bonds in the ring. Then, for each atom from the ring, angle alpha (see Figure S 1) was calculated. If any of these angles was out of range <85 ; 95> degrees, the ring was not classified as aromatic. Analogically, we calculated angle β for each of the substituent atoms directly connected to the ring. In this case the allowable range of values was <78 ; 102> degrees. Effectiveness of such an algorithm is almost 100 % (number of found aromatic aa/number of aromatic aa declared in mmcif files, see Figure S 1). Test was executed for cif files with resolution better or equal to 2.5 Å. For the worse quality structures effectiveness is approximately 97-98 % which is connected mainly with abnormal angles or length of bonds in such solutions.

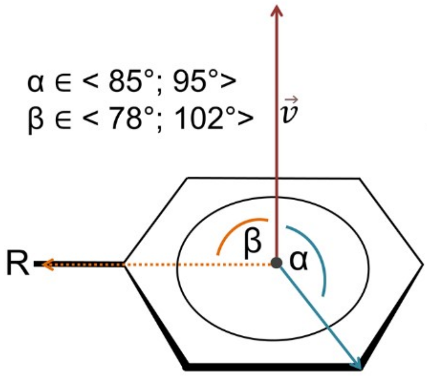

Amino acid	Detected aromatic rings	Number of occurrences	Effectiveness of detection [%]
PHE	2699404	2706570	99.7
HIS	1610110	1621174	99.3
TRP	1987366	995498	99.8
TYR	2369722	2376433	99.7

Figure S 1. Effectiveness of aromatic ring detection algorithm. The number of detected rings for tryptophan (TRP) is higher than the number of occurrences due to the presence of two rings in tryptophan residue.

Anions classification

Anion groups were searched inside a sphere centred at the ring centroid, with a radius of 5 Å. Search was focused on the following substructures: R-YOO$^-$ (Y = C, B), XO$^-$ (X = Cl, Br, I), XO_2^- (X = N, Cl, Br), XO_3^- (X = N, C, P, S, As, B, Cl, Br), XO_4^- (X = P, S, As, Cl, Br), R-OAO_3^- (A = P, S, As), R_2-OAO_3^- (A = P, S, As), R-AO_3^- (A = P, S, As), X^- (X = F, Cl, Br, I), SCN$^-$, CN$^-$, N_3^-. To identify such fragments we used the VF2 algorithm as implemented in the

networkx module.[11,12] To build graphs for the molecules in ring's neighbourhood we used the same criteria as described in section "Aromatic ring detection": 120% of the sum of atoms' valence radius was a threshold to identify these two atoms as bonded.

Cations classification

Every metal atom, treated as cation in our program, was searched for in the sphere with a radius of 10 Å of the ring's centroid. We also classified all nitrogen atoms from arginine and lysine with other names than "N" as positively charged (these were searched for in the sphere with a radius of 5 Å of the ring's centroid). We believe that this assumption is reasonable because $pK_{a,2}(\alpha\text{-}NH_3^+)$ of both ARG and LYS is usually close to 9, so in typical physiological pH those amino acids should be protonated.

Neighbouring ring

To find neighbouring rings might interact together, we use the same procedure as described in section "Aromatic ring detection". Parameters of all aromatic rings, which centres were closer than 5 angstroms from π-acid centroid were saved.

Hydrogen bonds

To analyse hydrogen bonds, coordinates of hydrogen atoms from structures were used if possible (NMR solutions, X-ray combined with neutron diffraction). In other cases hydrogen atoms were added to amino acids' oxygen and nitrogen atoms which were closer than 3.5 angstroms from anion atoms. For this purpose we slightly modified fragment of PropKa source code. Algorithm is based on VSEPR theory and was described by PropKa authors.[13–15] In the cases of hydroxyl group or 1° amine hydrogen atom added by our algorithm points into an anion, if possible (without interruption of sp^3 geometry).

Differences between coordinate systems

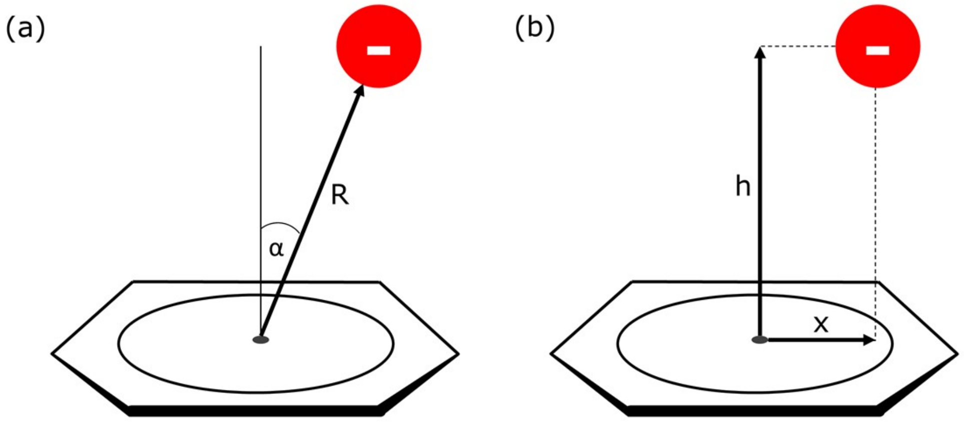

Figure S 2. Two different coordination systems (R,α) (a) and (h,x) (b) that might be used for the description of the position of chemical individuum in the space surrounding the aromatic species of interest.

R **and** ***α*** **coordinate system (*****R*****,*****α*****)**

Restriction used in the (R,α), coordinate system (R – distance between anion and ring centroid, α – angle between ring's normal vector and vector designated by anion and ring's centroid) lead to spherical cone shape of analysed space above aromatic ring. Analysis of anion distribution in function of R or α, should be carried out with caution. For instance, Lucas et all in their pioneering work[16] analysed anions in spherical cone designed by (2 < R < 5) Å and α < 45°. Histograms (number of anions in function of R or α) they presented indicate that number of anion grows with R and α without any maximum within the examined ranges. In fact in macromolecules from PDB local compaction of anion above the aromatic rings is observed. This contradiction is natural consequence of chosen coordinates - division of the analysed spherical cone into *equidistant* slices leads to *unequal* volume of compared slices (analogical for angle) (compare with Figure S 3 and equations below). The bigger analysed volume is, the bigger number of anions are found.

$$V = \frac{2}{3}\pi R^2 H = \frac{2}{3}\pi R^3 (1 - \cos \propto)$$ - volume of spherical sector

$$V_i(R) = \frac{2}{3}\pi H\left(R_i^{\,2} - R_{i+1}^{\,2}\right)$$

$$V_i(\propto) = \frac{2}{3}\pi R^3 (\cos \propto_i - \cos \propto_{i+1})$$

volume of i-slice of spherical sector

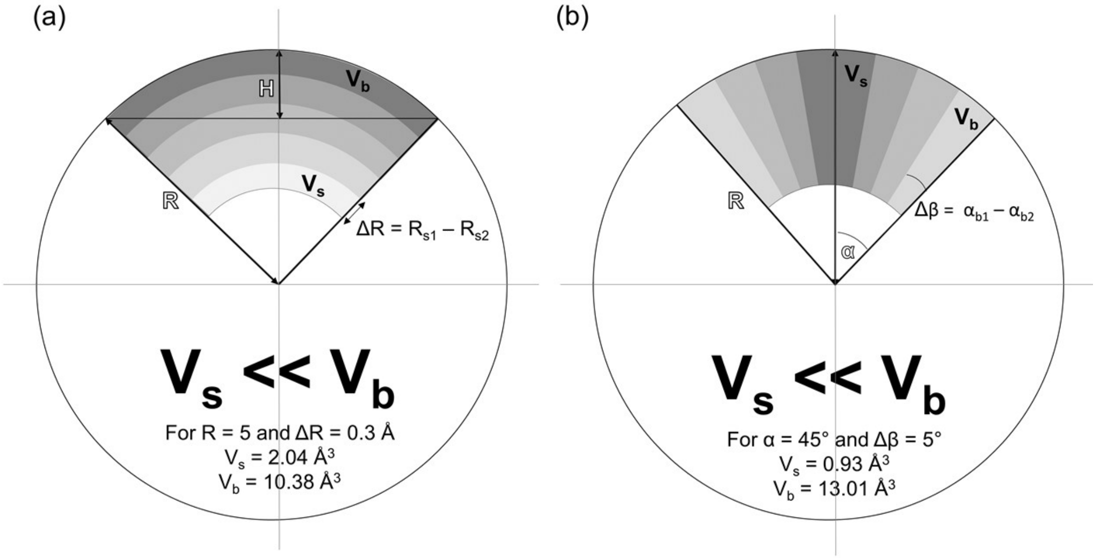

Figure S 3. Sliced spherical sector – cross-section. Subsequent slices have a different volume, and they shouldn't be compare without "normalization" in both (a and b) cases. Presented ratio of bins volume are related to histogram methodology presented by Lucas and co-workers.[16]

In our opinion the above immanent "feature" of (R,α) coordinate system should be stressed because R and α coordinates are widely used in analysis of chemical entity statistical distribution (including anions, cation, hydrogen bonds etc.), and although they are very useful, the authors of such research should be aware and bear in mind consequences of coordinate system they use.

h and *x* coordinate system (*x*,*h*)

The (x,h) coordinate system (x – distance from centroid in ring's plane, h – height over the ring plane) we used in our studies, allow us to note that above the aromatic rings local compaction of anion exists, assuming that x and h are measure to the closest atom of anionic group. Due to the fact that it is not the main location of anions (which prefer location in plane of the rings), and due to customary using of the R and α coordinates, this feature was overlooked until now.

$$V = \pi R^2 h$$ – volume of cylinder

$$\left.\begin{aligned} V_i(h) &= \pi R^2 (h_i - h_{i-1}) \\ V_i(x) &= \pi h (x_i^2 - x_{i-1}^2) \end{aligned}\right\}$$ volume of i-slice of spherical sector

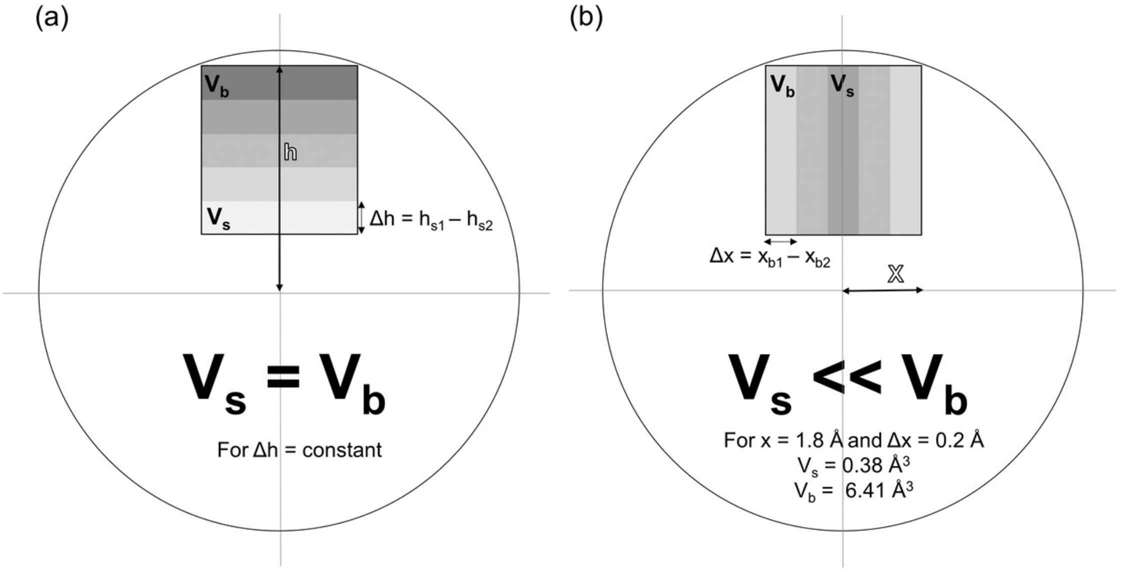

Figure S 4. Sliced cylinder – cross-section. If subsequent slices have the same height (Δh) therefore they the same volume (a) which can be compared.

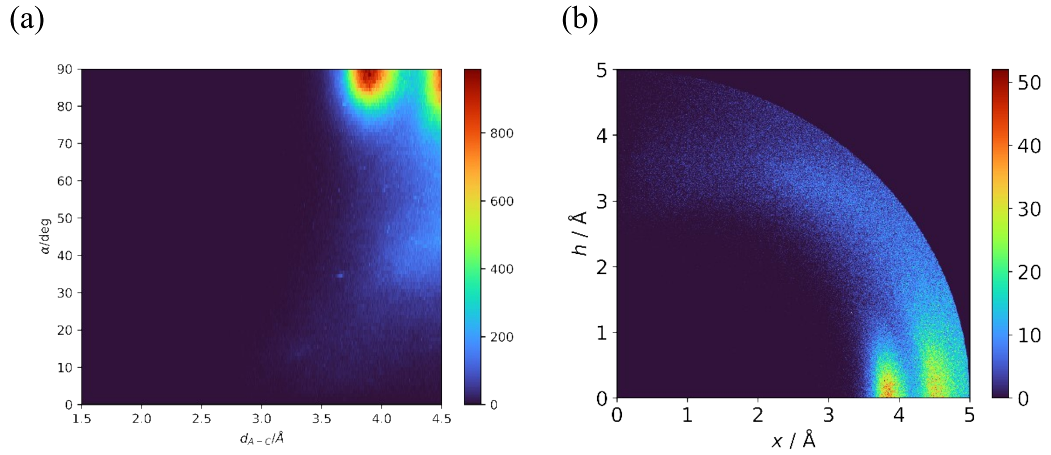

Figure S 5. Comparison of 2D histogram using (R,α) (a) or (x,h) (b) coordinate system. Histograms present distribution of anions obtained for uniqe pairs from structures with resolution better than 2.5Å.

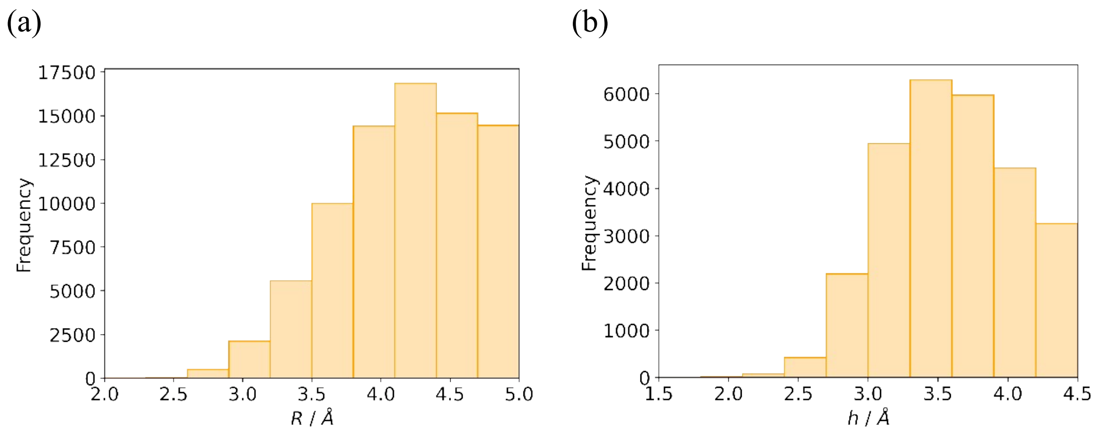

Figure S 6. Comparison of 1D histogram of R in spherical sector (a) and h in A region (b). In contrast to Lucas results[16], in our data set histograms of both R and h show local maximum. However, h histogram is more sharp and indicate that anions prefer location statistically closer to the rings. Histograms obtained for uniqe pairs from structures with resolution better than 2.5Å.

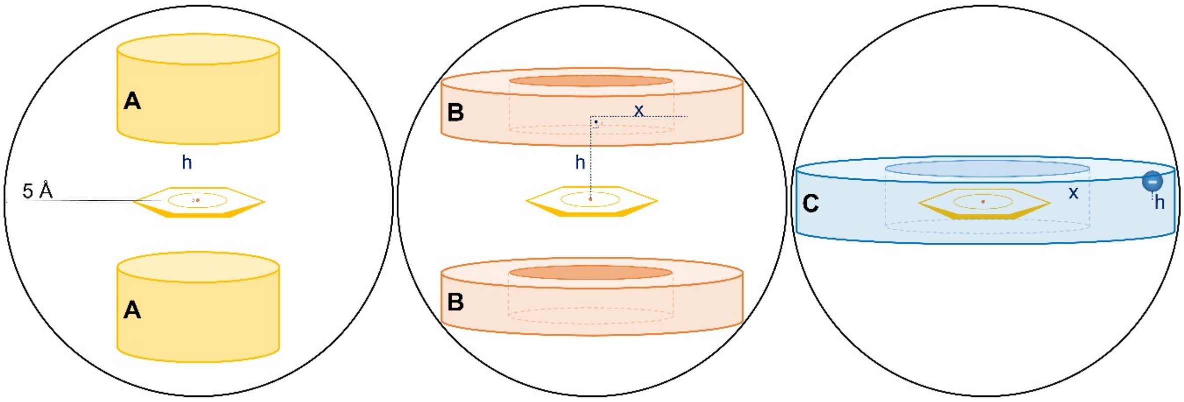

*Figure S 7. Schematic representation of shapes of the analysed region **A** (yellow), **B** (orange) and **C** (blue). Bins used to construction of 2D histogram of anion distribution and anion density have similar shape.*

Additional results

Aminoacids and nucleotides abbreviations are in common use. Their structures are presented in Figure S 19.

Ligands are introduced with their 3-letter codes and full name. To see their structures please visit http://ligand-expo.rcsb.org/ and use 3-letter code.

Histogram for all structures from PDB

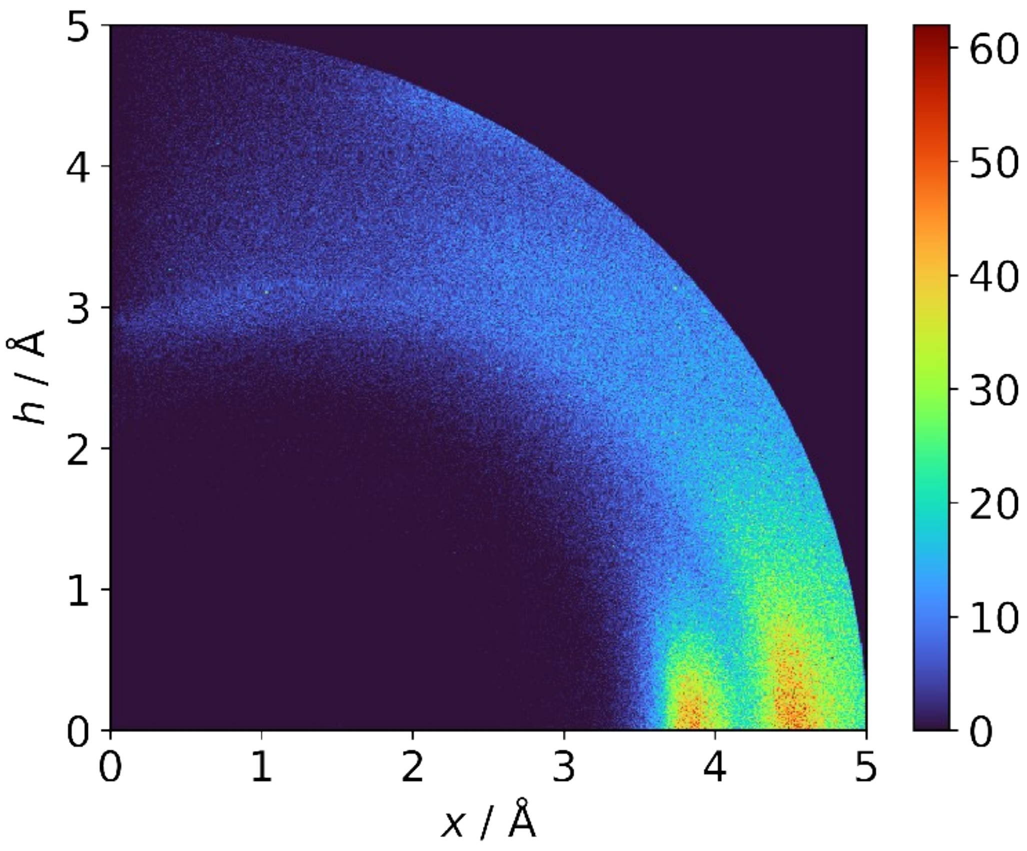

Figure S 8. Histogram for all structural models (independent of resolution and method).

Occurrences of anion-ring pairs - summary

Table S 1. Summary of unique anion-ring pair present in ***A*** *region of analysed macromolecules structures. Amino acid and sum of relative ribonucleotides and deoxyribonucleotides as quadrupole and as anion are presented in subsequent rows and columns. Abbreviations: APS - aspartic acid, GLU - glutamic acid, PHE, phenylalanine, TYR - tyrosine, HIS - histidine, TRP - tryptophan, A - sum of adenine ribonucleotide and deoxyribonucleotide, G - sum of guanine ribonucleotide and deoxyribonucleotide, T - sum of thymine ribonucleotide and deoxyribonucleotide, C - sum of cytosine ribonucleotide and deoxyribonucleotide, U - uracil ribonucleotide.*

Aromatic ring	Aminoacid as anion			Nucleotide as anion					Other molecules as anion	∑ anion-π interaction for ring
	ASP	GLU	other aa	A	G	T	C	U		
PHE	5164			48					1287	6499
	2365	2725	74	4	30	5	7	2		
TYR	5054			90					1519	6663
	2305	2636	113	43	10	9	26	2		
HIS	7909			76					1457	9442
	3724	4072	113	12	20	18	19	7		
TRP	3873			10					1144	5027
	1774	2030	69	1	3	3	2	1		
A	32			166					24	222
	14	17	1	66	50	1	23	26		
G	18			435					24	477
	8	10	0	235	128	2	35	35		
T	8			31					4	43
	4	4	0	1	20	4	6	0		
C	20			91					15	126
	15	4	1	33	24	0	14	20		
U	17			315					5	337
	7	10	0	128	93	0	40	54		
Others	1644			22					1048	2714
	705	929	10	6	9	0	1	6		
∑ anion-ring interaction for anion	10921	12437	381	529	387	42	173	153	6527	31550

Table S 2. Summary of unique anion-ring pair present in ***B*** *region of analysed macromolecules structures. Amino acid and sum of relative ribonucleotides and deoxyribonucleotides as quadrupole and as anion are presented in subsequent rows and columns.*

**Aromatic ring**	**Aminoacid as anion**			**Nucleotide as anion**					**Other molecules as anion**	**∑ anion-π interaction for ring**
	**ASP**	**GLU**	**other aa**	**A**	**G**	**T**	**C**	**U**		
**PHE**	17089			121					2070	19280
	6204	10702	183	31	25	23	36	6		
**TYR**	16661			242					2575	19478
	6226	10222	213	57	66	71	43	5		
**HIS**	20630			209					3457	24296
	8670	11762	198	47	58	30	54	20		
**TRP**	9727			55					1427	11209
	3573	6042	112	15	13	8	11	8		
**A**	107			311					39	457
	26	80	1	125	67	7	55	57		
**G**	53			403					44	500
	28	23	2	171	69	12	97	54		
**T**	29			19					13	61
	17	12	0	1	12	5	1	0		
**C**	55			173					40	268
	28	27	0	84	25	1	39	24		
**U**	11			187					14	212
	9	2	0	50	52	0	36	49		
**Others**	2998			27					1130	4155
	1528	1448	22	6	7	6	5	3		
**∑ anion-ring interaction for anion**	26309	40320	731	587	394	163	377	226	10809	79916

Table S 3. Summary of unique anion-ring pair present in ***C*** *region of analysed macromolecules structures. Amino acid and sum of relative ribonucleotides and deoxyribonucleotides as quadrupole and as anion are presented in subsequent rows and columns.*

Aromatic ring	Aminoacid as anion			Nucleotide as anion					Other molecules as anion	∑ anion-π interaction for ring
	ASP	GLU	other aa	A	G	T	C	U		
PHE	21436			272					2920	24628
	9540	11729	167	65	62	65	75	5		
TYR	75081			1233					10408	86722
	35729	38335	1017	302	388	207	268	68		
HIS	78076			1268					23392	102736
	39666	37416	994	358	358	225	260	67		
TRP	25713			400					4289	30402
	11946	13432	335	86	100	92	107	15		
A	92			388					54	534
	38	52	2	149	83	7	65	84		
G	397			1164					150	1711
	181	211	5	619	169	11	129	236		
T	74			14					7	95
	29	44	1	2	5	7	0	0		
C	318			654					80	1052
	152	161	5	264	125	14	133	118		
U	74			474					11	559
	31	39	4	156	145	0	85	88		
Others	10670			53					985	11708
	6602	4048	20	16	11	10	10	6		
∑ anion-ring interaction for anion	103914	105467	2550	2017	1446	638	1132	687	42296	260147

*Table S 4. Summary for most common anions. For each residue the unique occurrences in whole sphere were counted (sphere column), the occurrences of anion in region **A**, **B** and **C** together with the ratio of those occurrences. E. g. A/sphere = 6% means that 6 percent of anions located in close neighbourhood of ring, localize in region A.*

		**A**				**B**				**C**	
**Residue**	**sphere**	**A↓**	**A/sphere [%]**	**Residue**	**sphere**	**B↓**	**B/sphere [%]**	**Residue**	**sphere**	**C↓**	**C/sphere [%]**
GLU	203032	12190	6,0	GLU	203032	39386	19,4	GLU	203032	98488	48,5
ASP	177713	10805	6,1	ASP	177713	25670	14,4	ASP	177713	96711	54,4
SO4	14642	1099	7,5	SO4	14642	1704	11,6	SO4	14642	8477	57,9
ACT	4108	835	20,3	ACT	4108	857	20,9	CL	5963	2492	41,8
CL	5963	639	10,7	CL	5963	608	10,2	PO4	3920	2490	63,5
A	2984	468	15,7	PO4	3920	494	12,6	ACT	4108	1749	42,6
FMT	1866	435	23,3	A	2984	463	15,5	A	2984	1357	45,5
PO4	3920	337	8,6	HEM	1931	359	18,6	HEM	1931	1243	64,4
G	1870	302	16,1	FMT	1866	355	19,0	G	1870	727	38,9
ACY	736	172	23,4	G	1870	257	13,7	FMT	1866	696	37,3
HEM	1931	171	8,9	C	1413	241	17,1	U	1507	667	44,3
U	1507	153	10,2	CIT	775	228	29,4	DG	1278	658	51,5
C	1413	122	8,6	U	1507	224	14,9	DT	1186	612	51,6
NO3	718	107	14,9	NO3	718	166	23,1	DA	1055	559	53,0
CIT	775	92	11,9	ACY	736	163	22,1	DC	1093	534	48,9
MLI	376	85	22,6	DT	1186	161	13,6	CIT	775	528	68,1
IOD	894	84	9,4	DG	1278	131	10,3	C	1413	526	37,2
DG	1278	81	6,3	DC	1093	130	11,9	NAP	457	332	72,6
GLY	356	62	17,4	DA	1055	112	10,6	ACY	736	323	43,9
DA	1055	56	5,3	MES	593	112	18,9	NO3	718	297	41,4

*Table S 5. Summary for most common aromatic rings. For each residue the unique occurrences in whole sphere were counted (sphere column), the occurrences of anion in region **A**, **B** and **C** together with the ratio of those occurrences. E. g. A/sphere = 6% means that 6 percent of aromatic rings located in close neighbourhood of anion, have an anion above in region A.*

		**A**				**B**				**C**	
**Residue**	**sphere**	**A↓**	**A/sphere [%]**	**Residue**	**sphere**	**B↓**	**B/sphere [%]**	**Residue**	**sphere**	**C↓**	**C/sphere [%]**
HIS	143552	9138	6,4	HIS	143552	23169	16,1	HIS	143552	90269	62,9
TYR	150353	6440	4,3	TYR	150353	18827	12,5	TYR	150353	83139	55,3
PHE	88358	6284	7,1	PHE	88358	18700	21,2	TRP	62249	28887	46,4
TRP	62249	4854	7,8	TRP	62249	10804	17,4	PHE	88358	23834	27,0
G	2993	454	15,2	G	2993	397	13,3	G	2993	1446	48,3
U	1325	336	25,4	A	1659	376	22,7	C	1351	625	46,3
FAD	633	229	36,2	U	1325	207	15,6	U	1325	550	41,5
A	1659	196	11,8	C	1351	195	14,4	GDP	491	462	94,1
FMN	254	145	57,1	NAP	590	160	27,1	A	1659	445	26,8
NAD	1001	140	14,0	FAD	633	154	24,3	DC	708	400	56,5
NAP	590	135	22,9	NAD	1001	146	14,6	IMD	635	396	62,4
C	1351	113	8,4	HEM	323	106	32,8	PLP	443	386	87,1
ATP	242	73	30,2	DG	584	90	15,4	NAD	1001	262	26,2
HEM	323	54	16,7	IMD	635	71	11,2	DG	584	206	35,3
IMD	635	51	8,0	DA	325	69	21,2	NAP	590	197	33,4
DT	288	43	14,9	DC	708	69	9,7	GNP	196	190	96,9
NDP	252	36	14,3	DT	288	57	19,8	GTP	167	157	94,0
ADP	276	29	10,5	CLA	100	53	53,0	LLP	157	137	87,3
CLA	100	29	29,0	NDP	252	46	18,3	SAH	293	135	46,1
NAI	211	27	12,8	ADP	276	43	15,6	FAD	633	124	19,6
HEC	49	24	49,0	FMN	254	36	14,2	ADP	276	117	42,4

Anions distribution for the most common residues

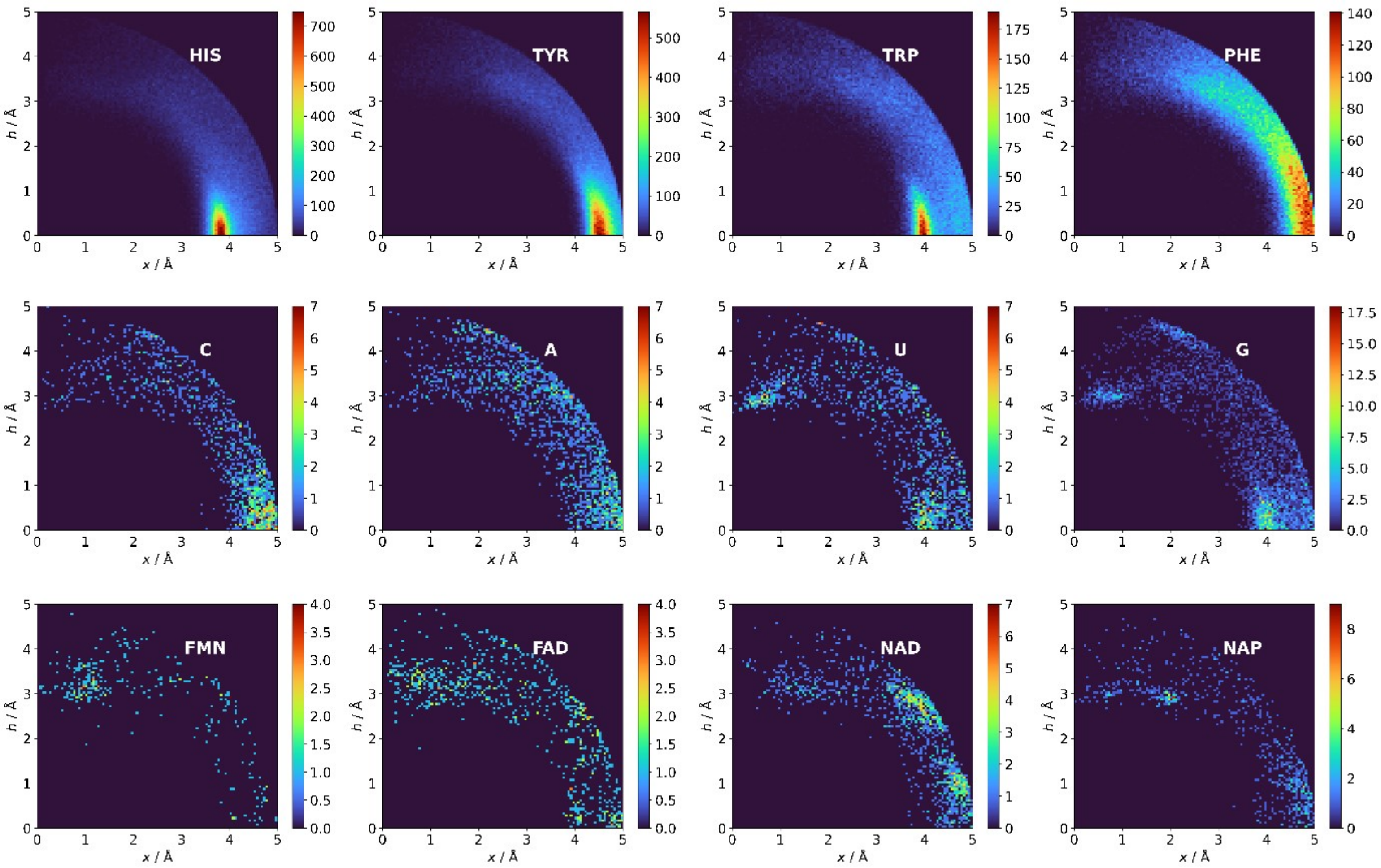

Figure S 9. 2D (x,h) histogram of the anions distribution for the selected aromatic rings. FAD - flavin-adenine dinucleotide, FMN - flavin mononucleotide, ATP - adenosine-5'-triphosphate, NAP - nicotinamide-adenine-dinucleotide phosphate.

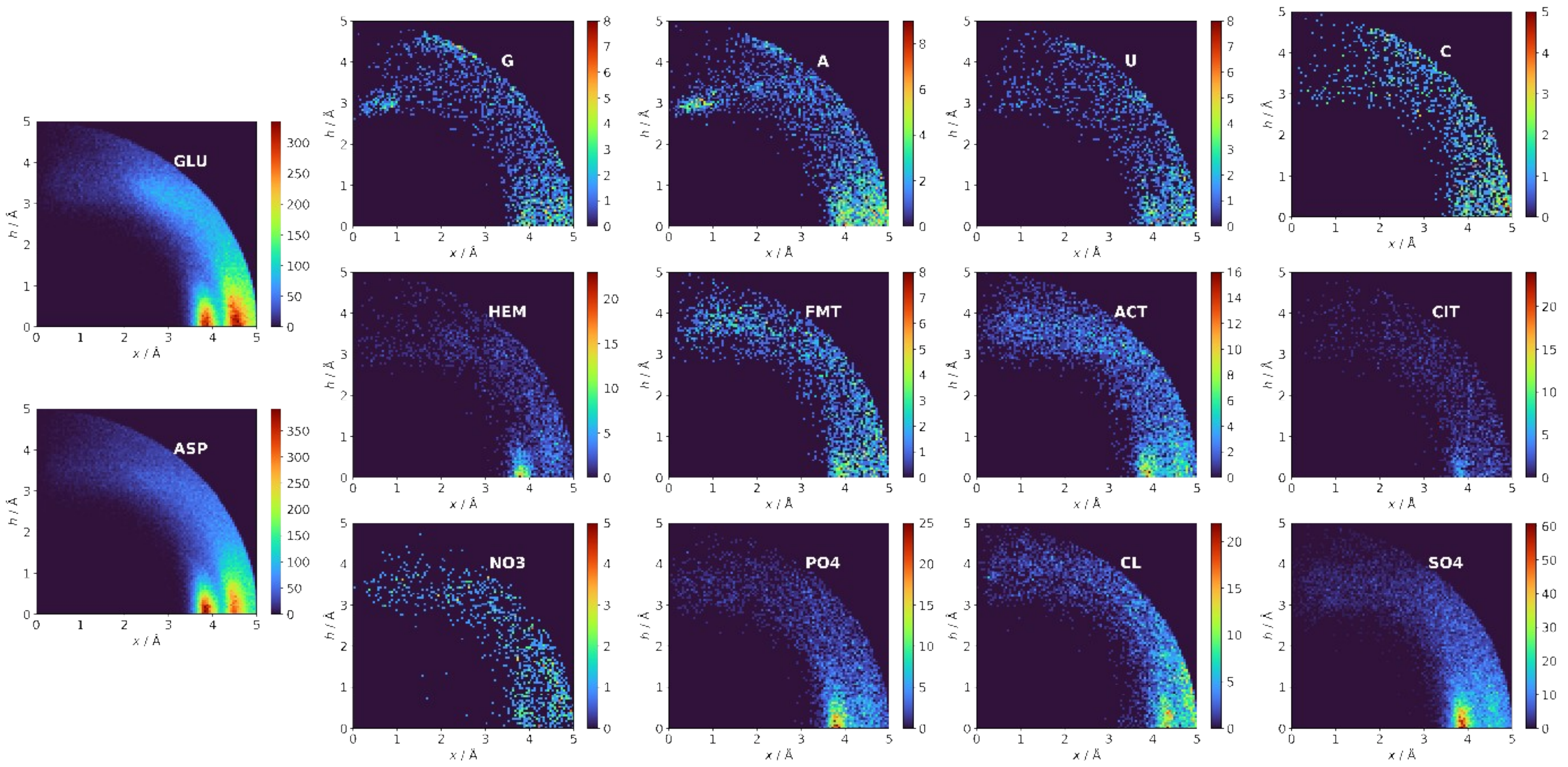

Figure S 10. 2D (x,h) of the selected anions distribution. HEM - protoporphyrin IX containing Fe, FMT - formic acid, ACT - acetate acid, CIT - citric acid, NO3 - NO_3^{-}, PO4 - PO_4^{3-}, CL – Cl^{-}, SO4 - SO_4^{2-}.

Structure type analysis

*Table S 6. Structure type distribution within regions **A**, **B** and **C**. In the table the number of structural files as well as number of found pairs in subsequent regions are given.*

	A			B			C		
	**Structural type**	**number**	**%**	**Structural type**	**number**	**%**	**Structural type**	**number**	**%**
**structural files**	protein	21928	95,77	protein	43476	96,39	protein	73466	96,17
	protein-DNA	537	2,35	protein-DNA	1034	2,29	protein-DNA	1969	2,58
	protein-RNA	261	1,14	protein-RNA	374	0,83	protein-RNA	604	0,79
	RNA	87	0,38	RNA	89	0,20	RNA	177	0,23
	protein-DNA-RNA	44	0,19	DNA	61	0,14	protein-DNA-RNA	103	0,13
	DNA	31	0,14	protein-DNA-RNA	59	0,13	DNA	65	0,09
	DNA-RNA	8	0,03	DNA-RNA	7	0,02	DNA-RNA	7	0,01
	other	1	0,00	other	2	0,00	other	1	0,00
**number of pairs**	protein	29364	93,07	protein	75976	95,07	protein	247835	95,26
	**protein-RNA**	**1222**	**3,87**	**protein-RNA**	**1812**	**2,27**	protein-DNA	6049	2,33
	protein-DNA	671	2,13	protein-DNA	1693	2,12	**protein-RNA**	**5209**	**2,00**
	**RNA**	**140**	**0,44**	protein-DNA-RNA	182	0,23	protein-DNA-RNA	554	0,21
	protein-DNA-RNA	96	0,30	**RNA**	**147**	**0,18**	**RNA**	**387**	**0,15**
	DNA	45	0,14	DNA	83	0,10	DNA	95	0,04
	DNA-RNA	11	0,03	DNA-RNA	19	0,02	DNA-RNA	17	0,01
	other	2	0,01	other	4	0,01	other	9	0,00

Sequence analysis

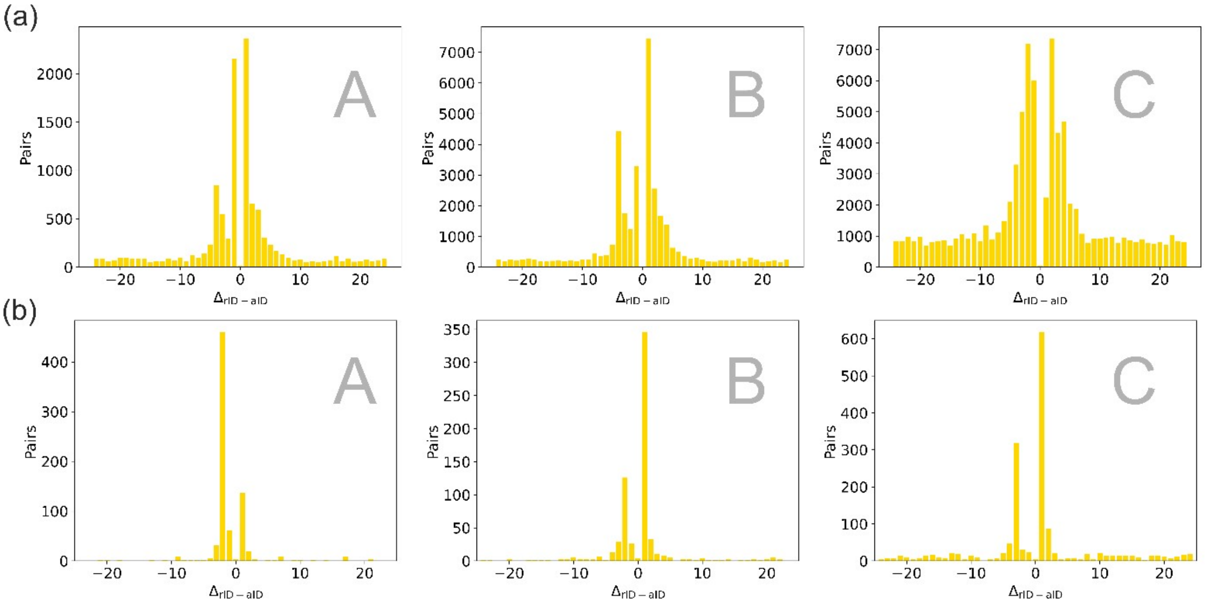

Figure S 11. Bar plots present differences between the ring and anion location in protein (a) or RNA (b) sequence (histograms for DNA and most common pairs are presented on Fig S 12-13). The difference between ring index and anion index (ΔrID-aID) indicates their order in the sequence. If the difference is positive the anion is located before a ring, otherwise the ring is before the anion. Data were not scaled by average amino acid frequency.[17]

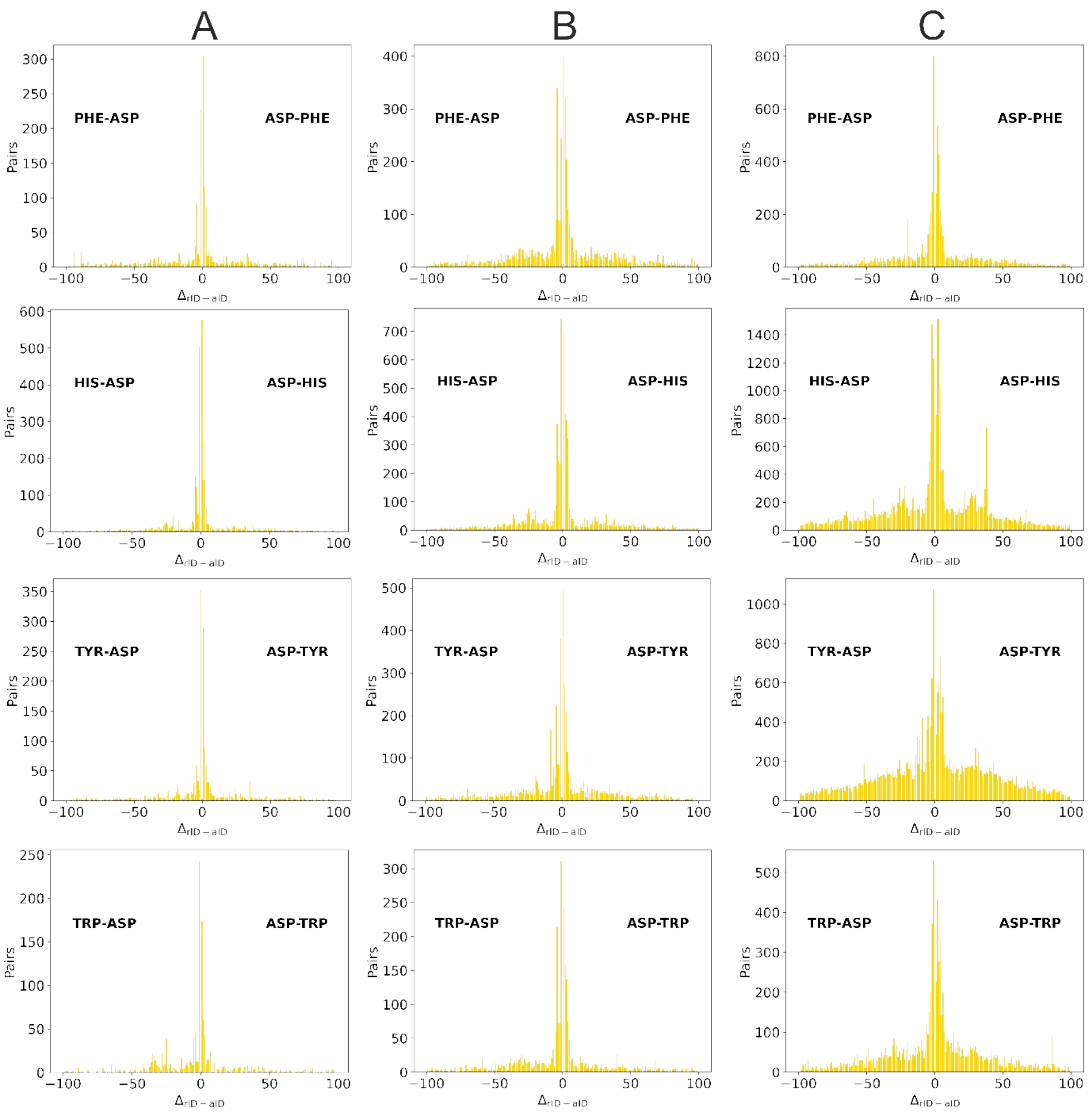

*Figure S 12. $\Delta_{rID\text{-}aID}$ distribution for Asp-Phe/His/Tyr/Trp pairs in region **A**, **B** and **C**. Data were not scaled by average amino acid frequency.*[17]

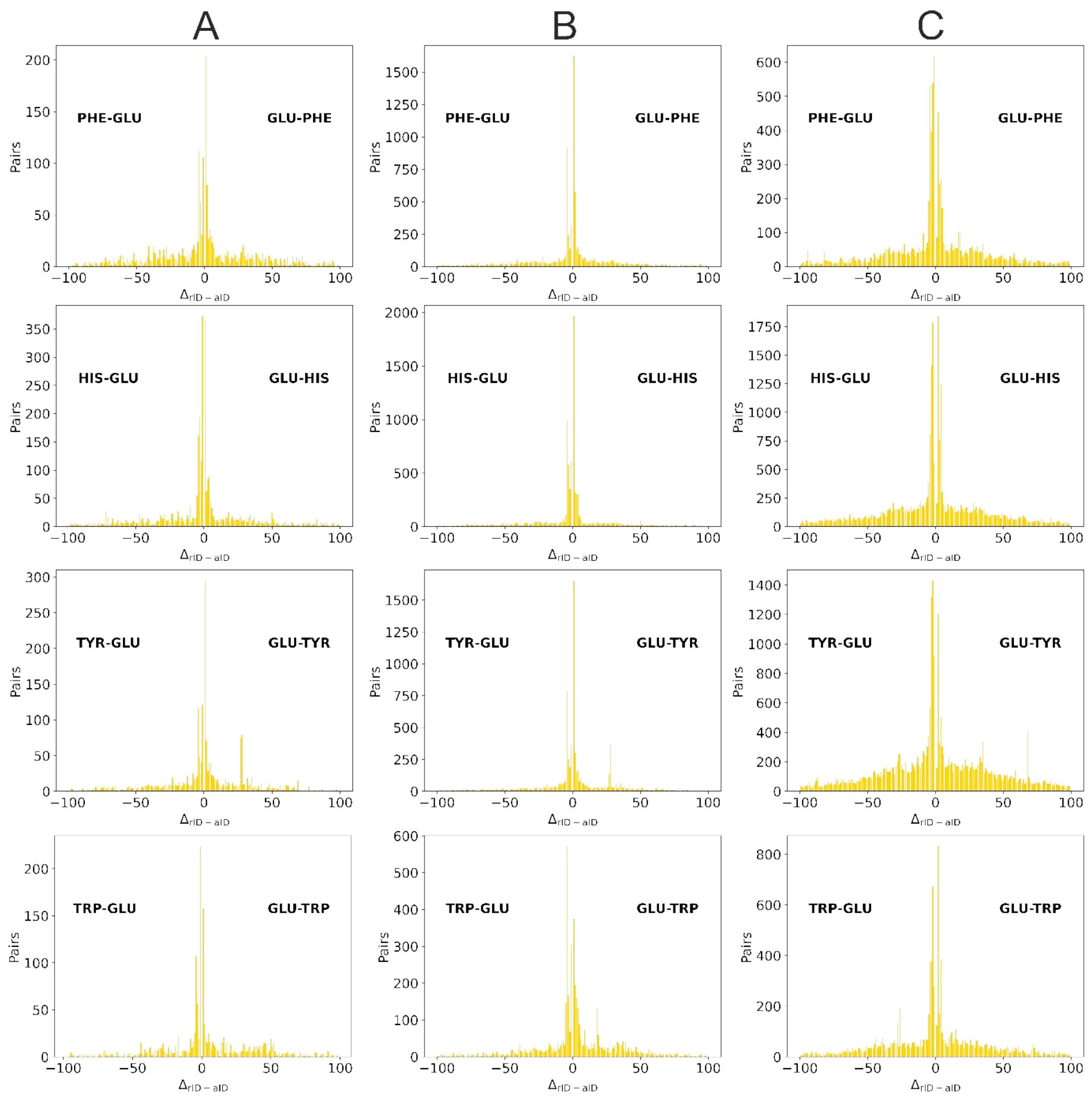

Figure S 13. $\Delta_{rID\text{-}aID}$ distribution for Glu-Phe/His/Tyr/Trp pairs in region A-C. Data were not scaled by average amino acid frequency.[17]

Table S 7. Secondary structure analysis (DSSP) of anion-ring pairs for different $\Delta_{rID\text{-}aID}$, molecules and regions A-C. E.g. in section Glu-Phe pairs $\Delta_{rID\text{-}aID} = 1$, (region A) both, anion and ring create an α-helix (H) which is 30,66 % of found cases.

**A**			**B**			**C**		
**ring**	**anion**	**%**	**ring**	**anion**	**%**	**ring**	**anion**	**%**
all aa no $\Delta_{rID\text{-}aID}$ restriction								
H	H	18,15	H	H	25,40	H	H	22,28
T	T	10,48	T	T	9,01	E	E	13,58
-	-	8,21	E	E	6,46	-	-	5,63
G	G	5,29	-	-	6,19			
			G	G	5,53			
all aa $\Delta_{rID\text{-}aID} = -1$								
T	T	25,76	T	T	22,45	H	H	72,97
E	T	13,68	H	H	15,57			
H	H	10,99	E	T	12,68			
G	G	9,80	G	G	11,13			
S	S	6,98	S	S	7,20			
-	-	6,25	-	-	7,17			
-	S	5,52						
-	T	5,11						
all aa $\Delta_{rID\text{-}aID} = -4$								
H	H	50,50	H	H	67,42	H	H	34,22
-	H	12,02	-	H	6,49	-	H	11,85
H	T	5,40	H	T	5,51	S	H	7,09
						-	-	5,38
all aa $\Delta_{rID\text{-}aID} = 1$								
H	H	22,60	H	H	21,79	H	H	13,73
T	T	12,68	T	T	19,32	S	S	12,58
G	G	9,38	G	G	13,10	T	T	10,44
-	-	7,83	S	S	8,38	G	G	7,73
S	S	7,74				E	E	7,07
-	S	5,40				-	S	6,80
Glu-Phe pairs $\Delta_{rID\text{-}aID} = 1$								
H	H	30,66	H	H	23,22	T	T	16,28
T	T	13,68	T	T	20,46	H	H	16,28
G	G	13,68	G	G	15,89	G	G	10,47
-	-	8,49	S	S	7,82	S	S	9,30
S	S	6,13	T	H	5,94	E	E	8,14
						-	S	6,98

**A**			**B**			**C**		
**ring**	**anion**	**%**	ring	anion	%	**ring**	**anion**	**%**
Glu-Phe/Tyr pairs $\Delta_{rID\text{-}aID} = 1$								
H	H	32,62	H	H	23,84	H	H	20,08
G	G	15,82	T	T	20,62	G	G	12,55
T	T	13,87	G	G	17,35	T	T	12,13
S	S	7,42	S	S	7,61	-	-	8,37
-	-	6,64	T	H	5,84	E	E	7,53
						S	S	6,69
						-	S	5,44
						S	T	5,44
Glu-Tyr pairs $\Delta_{rID\text{-}aID} = 1$								
H	H	34,00	H	H	24,45	H	H	22,22
G	G	17,33	T	T	20,78	G	G	13,73
T	T	14,00	G	G	18,77	T	T	9,80
S	S	8,33	S	S	7,40	-	-	9,80
-	-	5,33	T	H	5,75	E	E	7,19
						S	T	6,54

Planar anions orientation

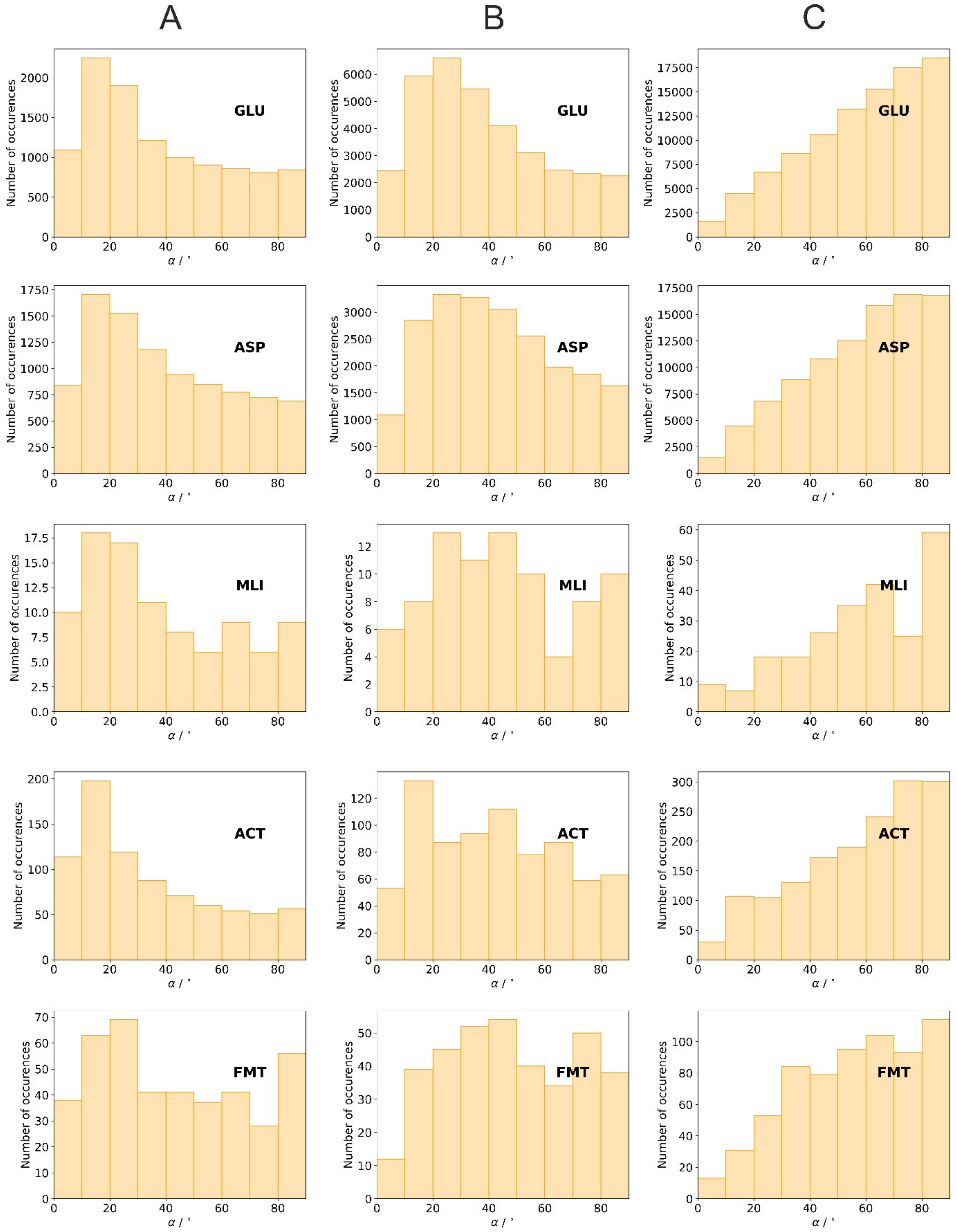

Figure S 14. Interplanar angle distribution. α = 0 correspond to the face-to-face geometry (both plane are parallel), whereas α =90 corresponds to the edgewise orientation. MLI – malonate ion, ACT – acetate ion, FMT – formic ion.

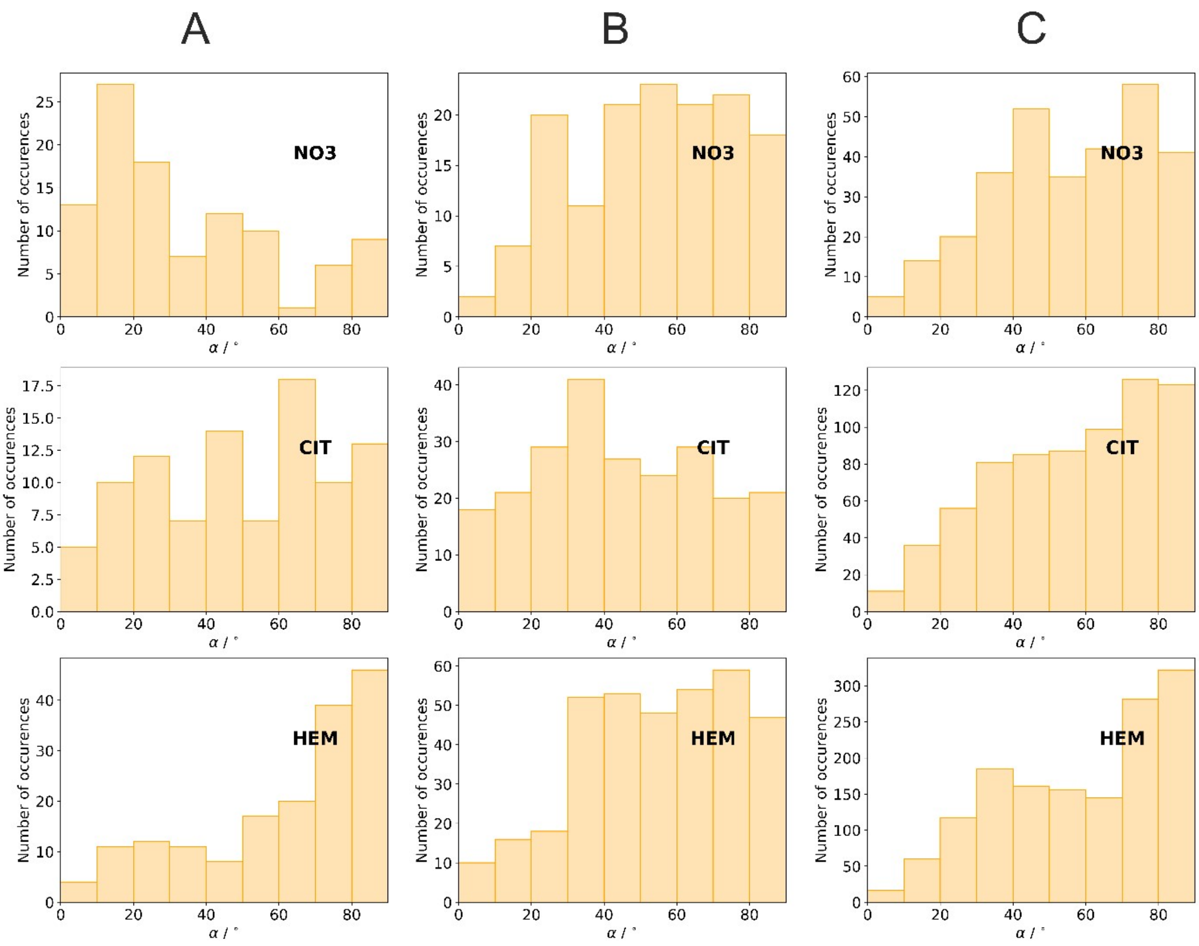

Figure S 15. Interplanar angle distribution – continuation.

Aromatics and other chemical entity around rings

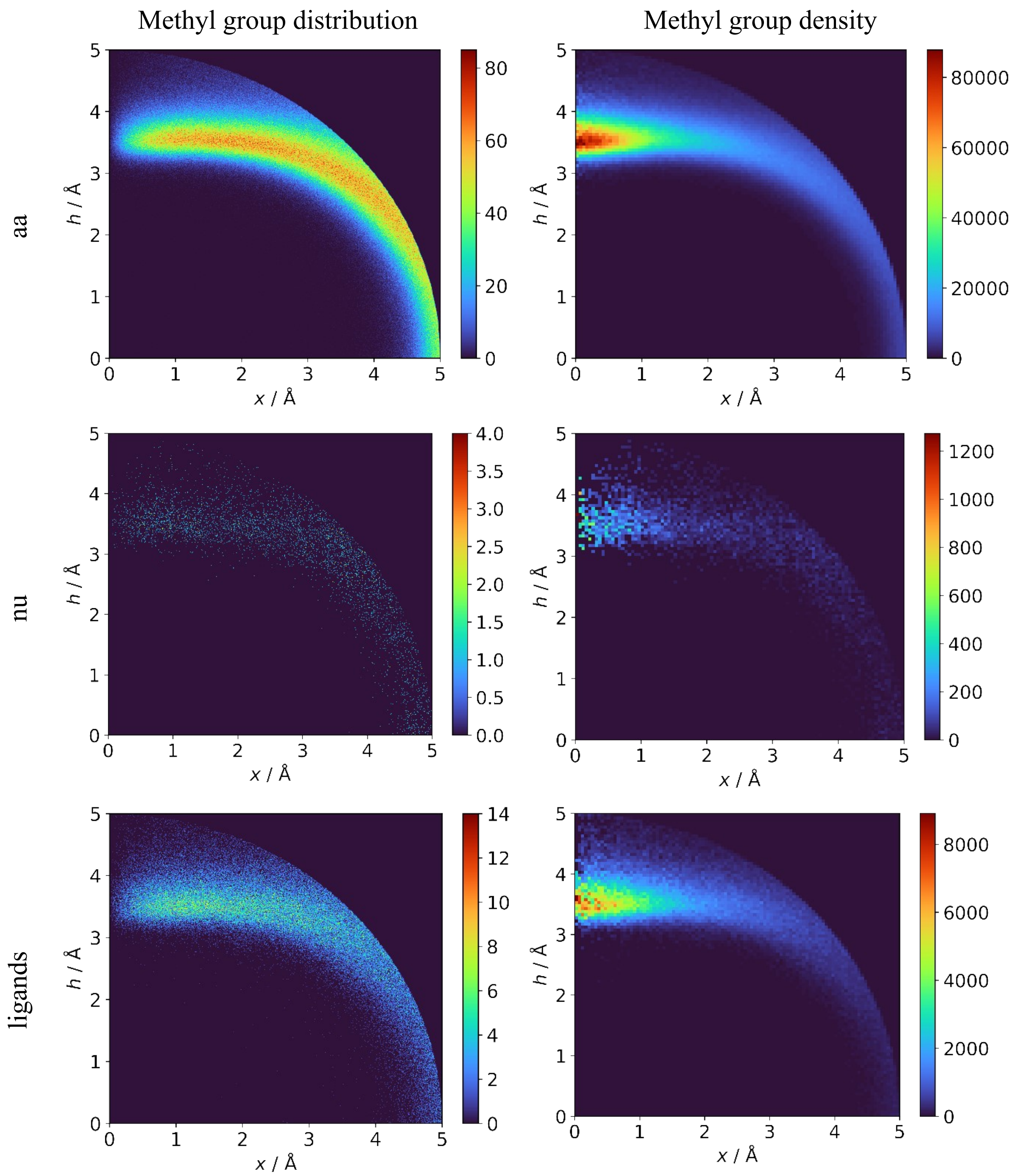

Figure S 16. Methyl groups distribution around aromatic rings in macromolecules.

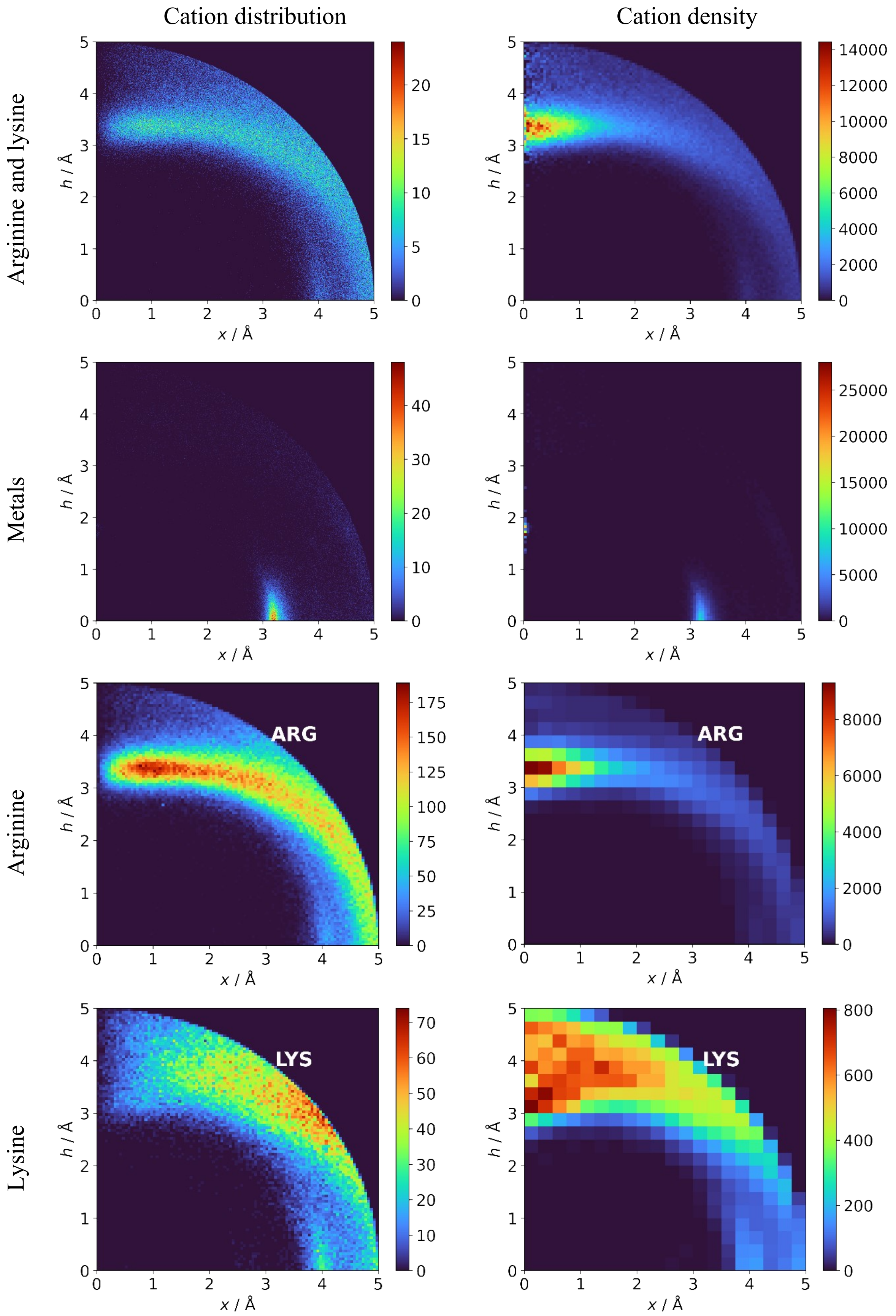

Figure S 17. Cations distribution around aromatic rings in macromolecules.

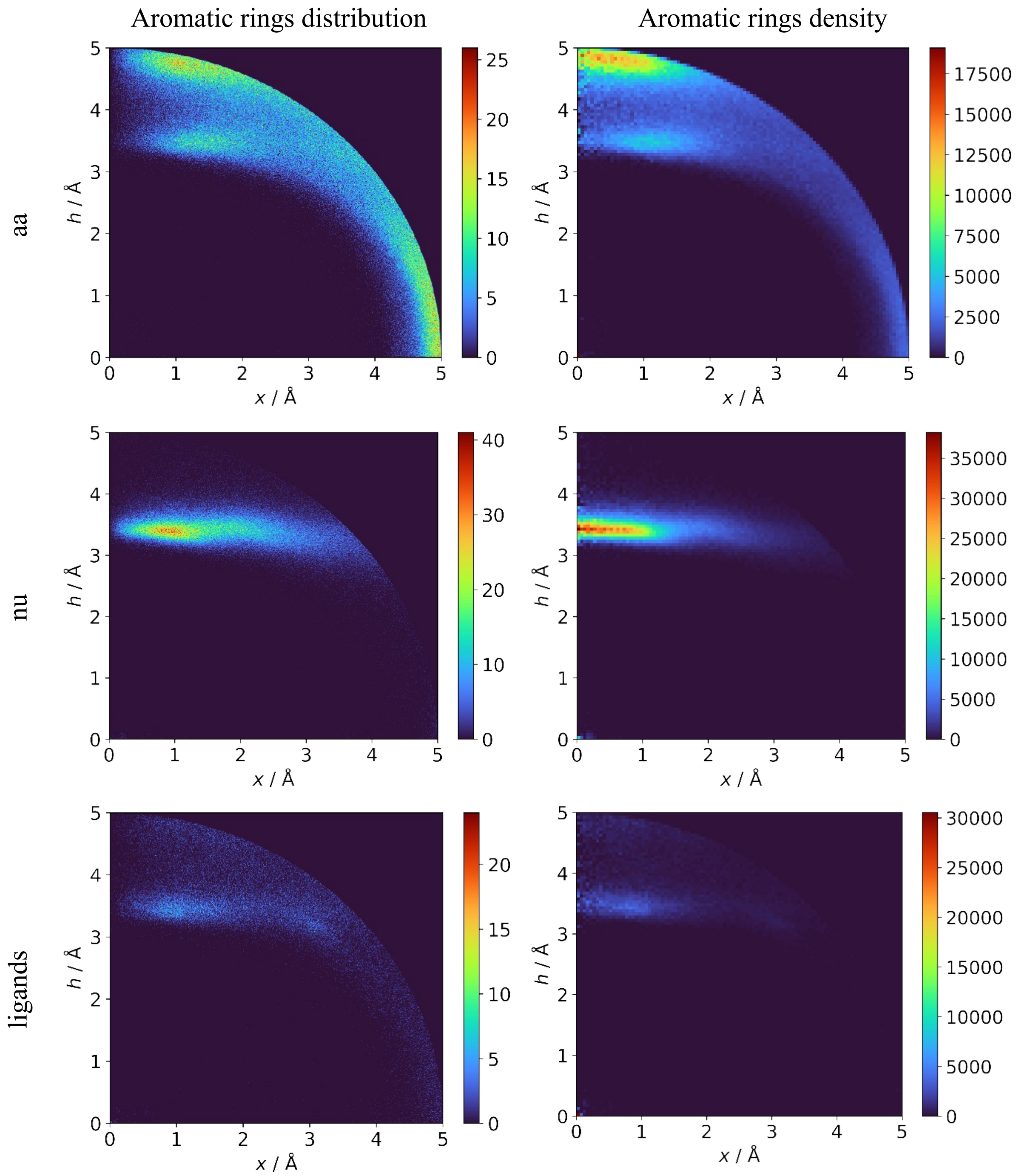

Figure S 18. Flat rings distribution around of aromatic rings in macromolecules.

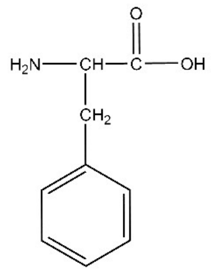

PHE

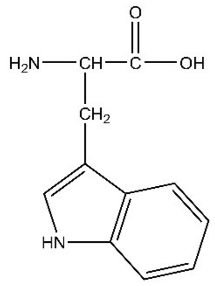

TRP

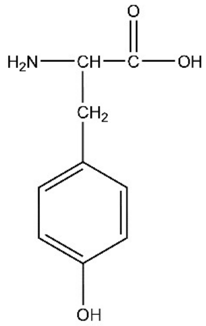

TYR

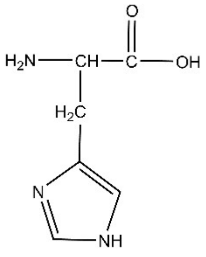

HIS

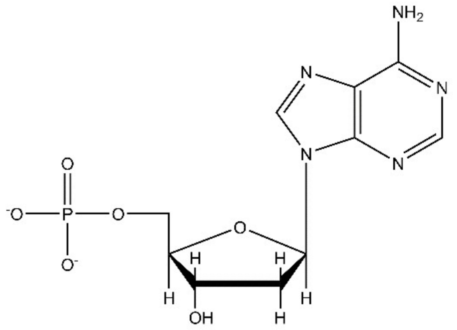

A

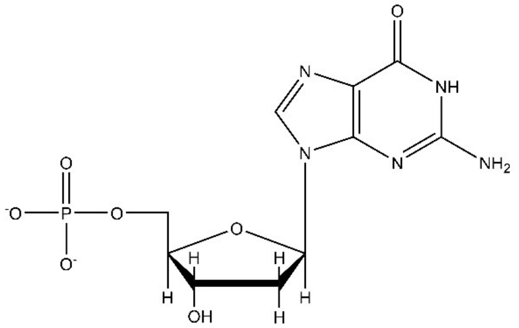

G

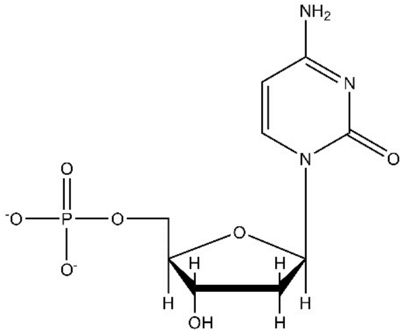

C

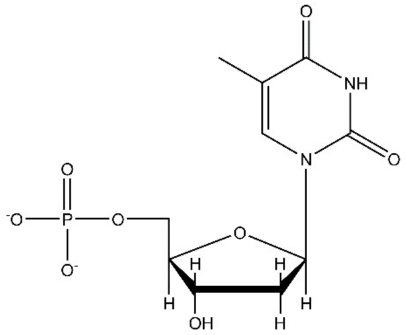

T

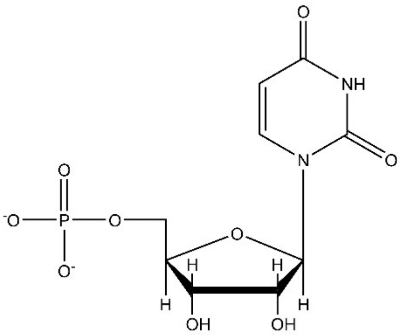

U

Figure S 19. Amino acids and nucleotides and their standard abbreviations used in PDB.

References

(1) Berman, H. M. The Protein Data Bank. *Nucleic Acids Res.* **2000**, *28* (1), 235–242. https://doi.org/10.1093/nar/28.1.235.

(2) Berman, H.; Henrick, K.; Nakamura, H. Announcing the Worldwide Protein Data Bank. *Nat. Struct. Mol. Biol.* **2003**, *10* (12), 980–980. https://doi.org/10.1038/nsb1203-980.

(3) Burley, S. K.; Bhikadiya, C.; Bi, C.; Bittrich, S.; Chen, L.; Crichlow, G. V; Christie, C. H.; Dalenberg, K.; Di Costanzo, L.; Duarte, J. M.; Dutta, S.; Feng, Z.; Ganesan, S.; Goodsell, D. S.; Ghosh, S.; Green, R. K.; Guranović, V.; Guzenko, D.; Hudson, B. P.; Lawson, C. L.; Liang, Y.; Lowe, R.; Namkoong, H.; Peisach, E.; Persikova, I.; Randle, C.; Rose, A.; Rose, Y.; Sali, A.; Segura, J.; Sekharan, M.; Shao, C.; Tao, Y.-P.; Voigt, M.; Westbrook, J. D.; Young, J. Y.; Zardecki, C.; Zhuravleva, M. RCSB Protein Data Bank: Powerful New Tools for Exploring 3D Structures of Biological Macromolecules for Basic and Applied Research and Education in Fundamental Biology, Biomedicine, Biotechnology, Bioengineering and Energy Sciences. *Nucleic Acids Res.* **2021**, *49* (D1), D437–D451. https://doi.org/10.1093/nar/gkaa1038.

(4) Hamelryck, T.; Manderick, B. PDB File Parser and Structure Class Implemented in Python. *Bioinformatics* **2003**, *19* (17), 2308–2310. https://doi.org/10.1093/bioinformatics/btg299.

(5) Cock, P. J. A.; Antao, T.; Chang, J. T.; Chapman, B. A.; Cox, C. J.; Dalke, A.; Friedberg, I.; Hamelryck, T.; Kauff, F.; Wilczynski, B.; de Hoon, M. J. L. Biopython: Freely Available Python Tools for Computational Molecular Biology and Bioinformatics. *Bioinformatics* **2009**, *25* (11), 1422–1423. https://doi.org/10.1093/bioinformatics/btp163.

(6) Li, W.; Godzik, A. Cd-Hit: A Fast Program for Clustering and Comparing Large Sets of Protein or Nucleotide Sequences. *Bioinformatics* **2006**, *22* (13), 1658–1659. https://doi.org/10.1093/bioinformatics/btl158.

(7) Fu, L.; Niu, B.; Zhu, Z.; Wu, S.; Li, W. CD-HIT: Accelerated for Clustering the next-Generation Sequencing Data. *Bioinformatics* **2012**, *28* (23), 3150–3152. https://doi.org/10.1093/bioinformatics/bts565.

(8) Pyykkö, P.; Atsumi, M. Molecular Single-Bond Covalent Radii for Elements 1-118. *Chem. - A Eur. J.* **2009**, *15* (1), 186–197. https://doi.org/10.1002/chem.200800987.

(9) Hagberg, Aric A.; Schult, Daniel A.; Swart, P. J. Proceedings of the Python in Science Conference (SciPy): Exploring Network Structure, Dynamics, and Function Using

NetworkX; Varoquaux G.; Vaught, T. . M. J., Ed.; 2008; pp 11–15.

(10) Paton, K. An Algorithm for Finding a Fundamental Set of Cycles of a Graph. *Commun. ACM* **1969**, *12* (9), 514–518. https://doi.org/10.1145/363219.363232.

(11) Cordella, L. P.; Foggia, P.; Sansone, C.; Vento, M. A (Sub)Graph Isomorphism Algorithm for Matching Large Graphs. *IEEE Trans. Pattern Anal. Mach. Intell.* **2004**, *26* (10), 1367–1372. https://doi.org/10.1109/TPAMI.2004.75.

(12) Cordella, L.P., Foggia, P., Sansone, C., Vento, M. An Improved Algorithm for Matching Large Graphs. In *In: Proc. 3rd IAPR –TC15 Workshop Graph Based Representations in Pattern Recognition*; 2001; pp 149–159.

(13) Olsson, M. H. M.; Søndergaard, C. R.; Rostkowski, M.; Jensen, J. H. PROPKA3: Consistent Treatment of Internal and Surface Residues in Empirical PKa Predictions. *J. Chem. Theory Comput.* **2011**, *7* (2), 525–537. https://doi.org/10.1021/ct100578z.

(14) Søndergaard, C. R.; Olsson, M. H. M.; Rostkowski, M.; Jensen, J. H. Improved Treatment of Ligands and Coupling Effects in Empirical Calculation and Rationalization of PKa Values. *J. Chem. Theory Comput.* **2011**, *7* (7), 2284–2295. https://doi.org/10.1021/ct200133y.

(15) Rostkowski, M.; Olsson, M. H.; Søndergaard, C. R.; Jensen, J. H. Graphical Analysis of PH-Dependent Properties of Proteins Predicted Using PROPKA. *BMC Struct. Biol.* **2011**, *11* (Cc). https://doi.org/10.1186/1472-6807-11-6.

(16) Lucas, X.; Bauzá, A.; Frontera, A.; Quiñonero, D. A Thorough Anion–π Interaction Study in Biomolecules: On the Importance of Cooperativity Effects. *Chem. Sci.* **2016**, *7* (2), 1038–1050. https://doi.org/10.1039/C5SC01386K.

(17) Meier, M.; Burkhard, P. Statistical Analysis of Intrahelical Ionic Interactions in α-Helices and Coiled Coils. *J. Struct. Biol.* **2006**, *155* (2), 116–129. https://doi.org/10.1016/j.jsb.2006.02.019.
